# Leukemia Inhibitory Factor Enhances Endogenous Cardiomyocyte Regeneration after Myocardial Infarction

**DOI:** 10.1371/journal.pone.0156562

**Published:** 2016-05-26

**Authors:** Masato Kanda, Toshio Nagai, Toshinao Takahashi, Mei Lan Liu, Naomichi Kondou, Atsuhiko T. Naito, Hiroshi Akazawa, Goro Sashida, Atsushi Iwama, Issei Komuro, Yoshio Kobayashi

**Affiliations:** 1 Department of Cardiovascular Medicine, Chiba University Graduate School of Medicine, Chiba, Japan; 2 Department of Cardiovascular Medicine, The University of Tokyo Graduate School of Medicine, Tokyo, Japan; 3 Department of Cellular and Molecular Medicine, Chiba University Graduate School of Medicine, Chiba, Japan; San Raffaele Pisana, ITALY

## Abstract

Cardiac stem cells or precursor cells regenerate cardiomyocytes; however, the mechanism underlying this effect remains unclear. We generated CreLacZ mice in which more than 99.9% of the cardiomyocytes in the left ventricular field were positive for 5-bromo-4-chloro-3-indolyl-β-d-galactoside (X-gal) staining immediately after tamoxifen injection. Three months after myocardial infarction (MI), the MI mice had more X-gal-negative (newly generated) cells than the control mice (3.04 ± 0.38/mm^2^, MI; 0.47 ± 0.16/mm^2^, sham; p < 0.05). The cardiac side population (CSP) cell fraction contained label-retaining cells, which differentiated into X-gal-negative cardiomyocytes after MI. We injected a leukemia inhibitory factor (LIF)-expression construct at the time of MI and identified a significant functional improvement in the LIF-treated group. At 1 month after MI, in the MI border and scar area, the LIF-injected mice had 31.41 ± 5.83 X-gal-negative cardiomyocytes/mm^2^, whereas the control mice had 12.34 ± 2.56 X-gal-negative cardiomyocytes/mm^2^ (p < 0.05). Using 5-ethynyl-2'-deoxyurinide (EdU) administration after MI, the percentages of EdU-positive CSP cells in the LIF-treated and control mice were 29.4 ± 2.7% and 10.6 ± 3.7%, respectively, which suggests that LIF influenced CSP proliferation. Moreover, LIF activated the Janus kinase (JAK)signal transducer and activator of transcription (STAT), mitogen-activated protein kinase/extracellular signal-regulated (MEK)extracellular signal-regulated kinase (ERK), and phosphatidylinositol 3-kinase (PI3K)–AKT pathways in CSPs *in vivo* and *in vitro*. The enhanced green fluorescent protein (EGFP)-bone marrow-chimeric CreLacZ mouse results indicated that LIF did not stimulate cardiogenesis via circulating bone marrow-derived cells during the 4 weeks following MI. Thus, LIF stimulates, in part, stem cell-derived cardiomyocyte regeneration by activating cardiac stem or precursor cells. This approach may represent a novel therapeutic strategy for cardiogenesis.

## Introduction

Progress in the treatment of heart failure has improved survival rates in previous decades; however, it remains one of the leading causes of morbidity and mortality worldwide [1]. Heart failure is caused by myocyte loss secondary to necrosis and/or apoptosis, which is complicated by adverse remodeling; many laboratories are investigating cardiac regenerative therapy designed to restore cardiomyocytes as a curative treatment. The most established strategy for cardiac regenerative therapy has been the delivery of exogenous cells, i.e., cell-based therapy. In the previous decade, many clinical trials have been conducted, which, in some cases, have demonstrated improved cardiac function [2,3]. However, the optimum cell types, the best preparation and delivery method, and the mechanisms underlying the beneficial action of the transplanted cells remain unclear [4].

A complementary regenerative strategy for cell-based therapy consists of the generation of new cardiomyocytes within the cardiac milieu to replace the injured myocardium. The heart tissue in some teleosts and amphibians is known to have high regenerative potential [5]. There are currently few clues regarding the regenerative potential of the mammalian heart; however, two important lines of evidence that suggest this ability have been reported. First, the adult mammalian heart possesses several types of cardiac stem cells (CSCs) or progenitor cells (CPCs), which have the ability to differentiate into cardiomyocytes [6]. Second, recent radioisotope studies have demonstrated homeostatic endogenous cardiomyocyte regeneration in the adult mammalian heart [7]. These findings support the possibility of enhancing the endogenous regeneration of heart tissue by stimulating the differentiation of dormant CSCs or CPCs.

Recent studies have demonstrated that several growth factors, cytokines, and chemicals may potentially enhance stem cell differentiation and engraftment following cell transplantation, promoting cardiac repair [8]. However, little is currently known regarding the factors that stimulate endogenous cardiac repair. We have previously reported that leukemia inhibitory factor (LIF) attenuates cardiac remodeling after myocardial infarction (MI) through anti-apoptotic and angiogenic effects [9]. Furthermore, we demonstrated that LIF increases the number of cardiomyocytes in the cell cycle and bone marrow (BM) cell-derived cardiomyocytes. LIF belongs to the interleukin-6 family of cytokines. The binding of LIF to its receptor initiates at least three distinct downstream signals, i.e., JAK–STAT, MEK–ERK, and PI3K–AKT [10]. LIF has opposite effects on different cell types at different developmental stages. LIF stimulates the proliferation of hematopoietic and neural progenitors, GB2 leukemic cells, and epidermal melanocytes, whereas it induces the differentiation of mesenchymal stem cells into kidney glomeruli and tubules, M1 leukemia, and breast cancer cells [11]. LIF is also required to maintain pluripotency in mouse embryonic stem (ES) cells; however, this function requires Oct-3/4, the expression of which is limited to ES and germ cells [12,13]. Thus, the multipotentiality-preserving action of LIF may not be prominent in adult organs. In muscle, LIF stimulates the proliferation of skeletal muscle satellite cells and induces cardiac myocyte hypertrophy, a growth response alternative to proliferation in terminally differentiated cells [14,15]. This pleiotropic feature of LIF implies that it is of interest to investigate the details of the association between LIF and cardiac regeneration via the proliferation or differentiation of CSCs and CPCs.

## Materials and Methods

### Ethics statement

All animal procedures were performed in accordance with the recommendations in the Guide for the Care and Use of Laboratory Animals of the National Institutes of Health. The protocol was approved by the Institutional Animal Care and Use Committee of Chiba University (project number 27–17). Animals were fed ad libitum with standard chow and water in the institutional specific pathogen-free housing. Anesthesia (Isoflurane) was used during surgery and at the time of sacrifice. All efforts were made to minimize the suffering of the animals.

### Animals

**Fig 1:**
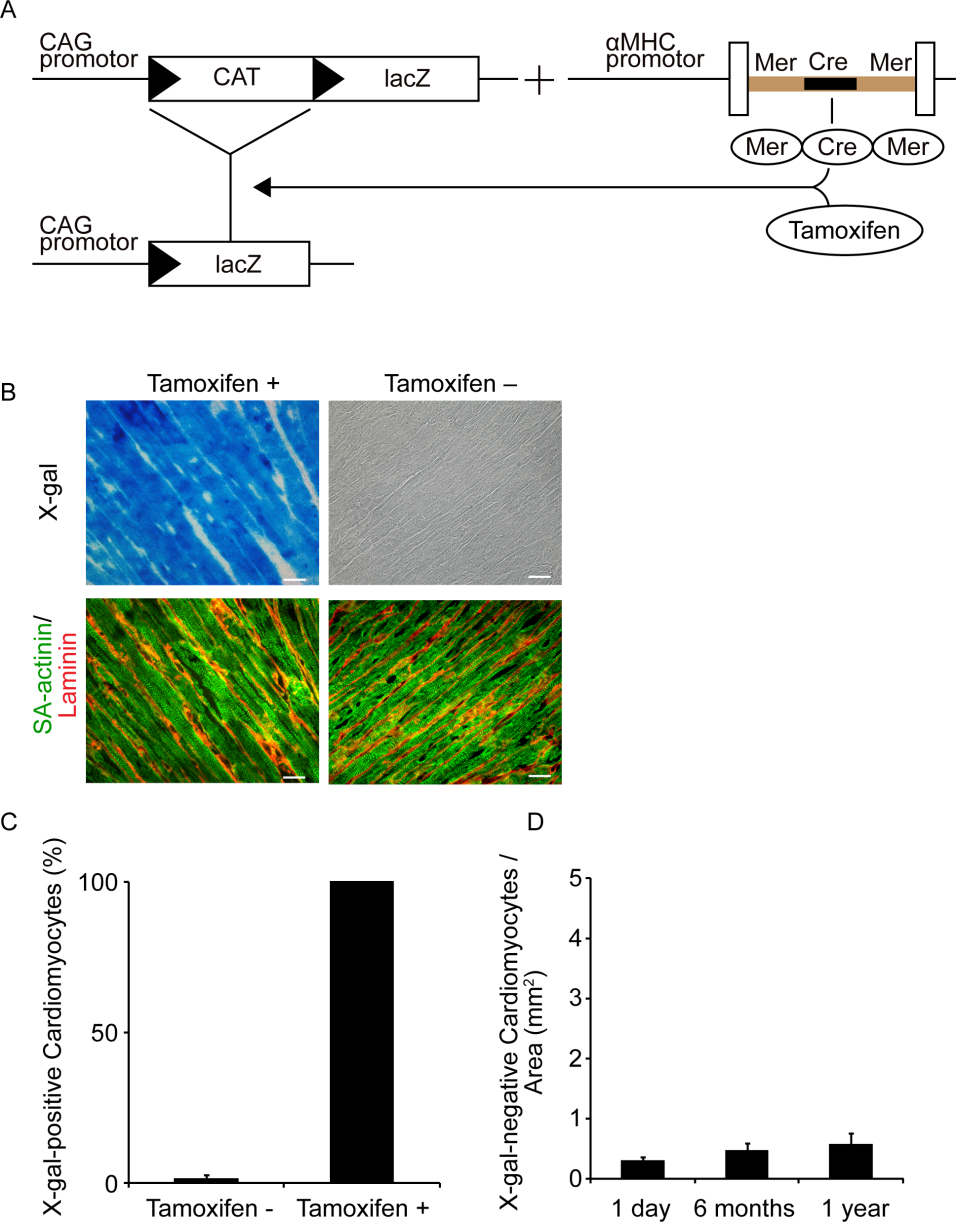
Efficiency and Specificity of Tamoxifen-induced β-galactosidase Expression in Cardiomyocytes in CreLacZ Mice. (A) Generation of CreLacZ mice. CAG-CAT-LacZ mice were crossbred with the MerCreMer strain in which tamoxifen-inducible Cre recombinase expression protein was driven by the α-myosin heavy chain promoter. (B) β-galactosidase expression in cardiomyocytes detected by X-gal staining in a tamoxifen-treated CreLacZ mouse (left) and non-treated mouse (right). The bottom panels show immunofluorescence images of the same samples co-stained with sarcomeric α-actinin (SA-actinin) in green and laminin in red. Scale bar, 20 μm. (C) Frequency of X-gal-positive cardiomyocytes in the LV with or without tamoxifen administration (n = 1833 cardiomyocytes pooled from three sections from three mice and n = 1538 cardiomyocytes pooled from two sections from two mice, respectively). The mice were examined immediately after completion of tamoxifen treatment. (D) Number of X-gal-negative cardiomyocytes per area in CreLacZ mice at 1 day (n = 5), 6 months (n = 5), and 1 year (n = 4) after tamoxifen treatment. The Kruskal–Wallis test showed no significant difference among the three groups.

Double-transgenic mice (CreLacZ mice) were obtained by crossbreeding transgenic mice. The first strain harbored tamoxifen-inducible Cre recombinase proteins fused to two mutant estrogen-receptor ligand-binding domains (MerCreMer), which were expressed under the control of the α-myosin heavy chain promoter (provided by Dr. Jeffery D Molkentin, Cincinnati Children’s Hospital Medical Center, Cincinnati, OH, USA) [16]. These mice were crossbred with mice that harbored the cytomegalovirus immediate early enhancer chicken β-actin hybrid promoter (CAG)-chloramphenicol acetyl transferase (CAT)-LacZ, in which β-galactosidase is expressed following the removal of a loxP-flanked stop sequence (provided by Dr. Jyunichi Miyazaki, Osaka University Graduate School of Medicine, Osaka, Japan) (Fig 1A) [17]. Cre recombination was induced by intraperitoneally (ip) injecting CreLacZ mice daily with tamoxifen (T5648, Sigma–Aldrich, St Louis, MO, USA), which was dissolved at 4 mg/mL in corn oil (C8267, Sigma–Aldrich), at a dosage of 5 mg/kg body weight for 14 consecutive days. To validate the cardiomyocyte-specific β-galactosidase expression, sections of CreLacZ mouse hearts were subjected to immunostaining for sarcomeric α (SA)-actinin and laminin in conjunction with 5-bromo-4-chloro-3-indolyl-β-D-galactopyranosidase (X-gal) staining. This treatment induces cardiotoxicity in MerCreMer mice [18]; however, the low-dose administration protocol followed herein caused no significant cardiac dysfunction in the CreLacZ mice (S1 Table). All strains were maintained on a C57BL/6J genetic background after backcrossing. Genotyping was performed by polymerase chain reaction (PCR) using tail DNA with the following primers: MerCreMer forward, 5´-GTTCGCAAGAACCTGATGGACA-3´; MerCreMer reverse, 5´-CTAGAGCCTGTTTTGCACGTTC-3´; LacZ forward, 5´-CCTTTCGCCAGCTGGCGTAATAGCG-3´; and LacZ reverse, 5´-ACCGTGCATCTGCCAGTTTG-3´.

Ten- to fifteen-week-old (at the time of tamoxifen administration) double-heterozygous CreLacZ mice were used for the experiments. C57BL/6J and enhanced green fluorescent protein (EGFP) transgenic mice were purchased from Sankyo Labo Service Corp. (Tokyo, Japan).

### EGFP chimeric mice

Whole bone marrow cells (3 × 10^6^) from transgenic EGFP mice were intravenously injected into lethally (8.5-Gy) irradiated 10-week-old CreLacZ mice [19]. Four weeks after bone marrow transplantation, peripheral blood samples were collected from the recipient mice. Donor bone marrow-derived cells were identified by the EGFP fluorescence intensity, and mice engrafted with more than 95% EGFP-positive cells were used for the experiments.

### Animal surgery

Three days after the 14th tamoxifen injection, we subjected the MerCreMer-LacZ mice to sham or experimental myocardial infarction (MI) conditions. MI was produced by a permanent ligation of the left coronary artery 3 mm below the left atrial appendix [9].

### Immunohistochemistry and immunofluorescence

Fresh-frozen sections were fixed with 4% paraformaldehyde for 10 min at room temperature. For p-AKT and p-ERK staining, cells were permeabilized with methanol for 10 min at -20°C between fixation and pre-blocking. After pre-blocking with phosphate-buffered saline (PBS) containing 2% donkey serum (017-000-121, Jackson ImmunoResearch Laboratory, West Grove, PA, USA), 2% bovine serum albumin (A7888, Sigma−Aldrich), and 0.2% Nonidet P-40 (25223–04, Nacalai Tesque, Kyoto, Japan) for 30 min, primary antibodies in PBS containing 2% donkey serum, 2% bovine serum albumin, and 0.1% Nonidet P-40 were added and incubated overnight at 4°C. The samples were subsequently washed three times in PBS, and Alexa 488-, Cy3-, or Cy5-conjugated secondary antibodies were applied to visualize the expression of specific proteins. Nuclear staining was performed with TO-PRO-3 (T3605, Molecular Probes, Eugene, OR, USA) or DAPI (D9542, Sigma–Aldrich). For double immunostaining of Sca-1 and GATA4, fresh-frozen sections were fixed with cold acetone, and detergent treatment was omitted. For 5-bromo-2′-deoxyurinide (BrdU) detection, 12% HCl was applied for 10 min, and primary anti-BrdU antibody was applied. For Nkx2.5 detection, a tyramide signal amplification system (T20925, Life Technologies, Carlsbad, CA, USA) was used according to the manufacturer’s instructions. To examine β-galactosidase expression, the samples were incubated for 20 h after secondary antibody incubation in a 0.1 mM phosphate buffer (pH 7.3) solution of 1 mg/mL X-gal chromagen (9031, TaKaRa, Osaka, Japan), 5 mM K_3_Fe(CN)_6_, 5 mM K_4_Fe(CN)_6_, 2 mM MgCl_2_, 0.01% sodium deoxycholate, 0.01% NP40, and 20 mM Tris-HCl (pH 7.3). The morphology of the heart sections was examined using hematoxylin (SFJ 8650, Sakura Finetek Japan, Tokyo, Japan) and eosin (SFJ8659, Sakura Finetek Japan) staining. The MI fibrous area was visually identified by Masson’s trichrome staining (4035–2, Muto Pure Chemicals Co., Ltd, Tokyo, Japan). The infarct size is expressed as the percentage of the fibrous area divided by the whole area.

The number of regenerated cardiomyocytes in the PBS- and LIF-treated CreLacZ mice was estimated as follows. Short-axis and longitudinally oriented X-gal-negative cardiomyocytes in the heart section occupy a region equivalent to its cell length and width in the direction orthogonal to the section, respectively, and the frequency of X-gal-negative cardiomyocytes is constant in the MI border and scar area; thus, the total number of X-gal-negative cardiomyocytes in the MI border and scar area, N, was calculated as follows:

N=R/l×A×nshort+R/w×A×nlong,

where R indicates the outer diameter of the left ventricle (LV) and where l and w are the myocyte length and width, respectively, A indicates the area of the MI border and scar obtained by digital planimetry, and nshort and nlong are the numbers of short-axis and longitudinally oriented X-gal-negative cardiomyocytes per mm^2^ of a section, respectively. We defined l as 80 μm and w as 20 μm, as previously reported [20].

### Cardiac side population (CSP) cell isolation

CSPs were isolated as previously described [21]. The minced cardiac tissue from freshly isolated mouse hearts was digested using collagenase type 2 (LS004174, Worthington Biochemical Corp, Lakewood, NJ, USA) and dispase (17105–041, Life Technologies). The cells were resuspended at a density of 1.0 × 10^6^/mL in PBS containing 3% fetal bovine serum. The cells were incubated in 1 μg/mL Hoechst 33342 dye (H3570, Molecular Probes) for 60 min at 37°C in the dark, with or without 50 μM verapamil. Following incubation, the cells were analyzed using Altra flow cytometric analysis (Beckman Coulter, Fullerton, CA, USA). Hoechst 33342 dye was excited at 350 nm using an ultraviolet laser. Fluorescent emission was detected using 450-nm bandpass (Hoechst blue) and 675-nm longpass (Hoechst red) filters, respectively. Isolated CSPs were used for in vitro incubation experiments or directly centrifuged onto slides using a CytoSpin Cytocentrifuge (Thermo Fisher Scientific, Waltham, MA, USA) at 71.8 × g for 5 min. For the β-galactosidase expression analysis, isolated CSPs and residual cardiomyocytes were fixed with 0.25% glutaraldehyde for 10 min and stained in X-gal staining solution for 1 h. For normal immunofluorescence staining, CSPs were fixed with 4% paraformaldehyde for 10 min and stained using the method described for fresh-frozen section staining.

### Cardiomyocyte isolation and flow cytometry with fluorescein di-β-D-galactopuranoside (FDG)

Cardiomyocytes and other cells were isolated from the ventricular areas of CreLacZ mice immediately after completion of tamoxifen treatment using a modification of a previously described protocol [22]. The heart was digested with collagenase type 2 for 15 min. The isolated cells were stained with the FluoReporter^®^ lacZ Flow Cytometry Kit (F-1930, Life Technologies, Carlsbad, CA, USA) according to the manufacturer’s instructions and analyzed using EPICS Altra flow cytometric analysis. β-galactosidase was detected via the expression of FDG. Fluorescein emission was detected through a 525-nm bandpass filter. Propidium iodide (PI) was used to analyze cell viability. The isolated cells were sorted according to their emission level and directly centrifuged onto slides using a CytoSpin Cytocentrifuge at 71.8 × g for 5 min. For α-MHC staining and X-gal staining, the cells were fixed with 0.25% glutaraldehyde for 10 min and stained in X-gal staining solution for 3 h.

### Injection of plasmids expressing LIF cDNA

The LIF expression vector was constructed by the insertion of a chimera of human and mouse LIF cDNA into the pCAGGS plasmid, which is driven by both a cytomegalovirus enhancer and a chicken α-actin promoter [9]. The plasmid was prepared using the PowerPrep Plasmid Purification Kits (NP100008, OriGene Technologies, Inc., Rockville, MD, USA) and was injected at a dose of 100 μg plasmid in 100 μL PBS per 20 g body weight into the thigh muscle of the mice immediately after MI induction. The same volume of PBS alone was injected into littermates as controls.

### Antibodies

The primary antibodies included mouse monoclonal anti-SA-actinin (1:200, A7811, Sigma–Aldrich), mouse BA-G5 monoclonal antibody specific for cardiac α-myosin heavy chain (1:100, ab50967, Abcam, Cambridge, MA, USA), rabbit polyclonal anti-laminin (1:200, L9393, Sigma–Aldrich), mouse monoclonal anti-beta-galactosidase (1:100, 0863363, MP Biomedicals, Santa Ana, CA, USA; LLC-Cappel), rat monoclonal anti-laminin-α-2 (1:200, sc-59854, Santa Cruz Biotechnology, Dallas, TX, USA), rat monoclonal anti-BrdU (1:250, OBT0030, AbD Serotec, Raleigh, NC, USA), mouse monoclonal anti-multi-drug-resistant protein 1 (MDR1) (1:50, ALX801-002-C100, Alexis, Lausen, Switzerland), rabbit polyclonal anti-Nkx2.5 (1:500, ab35842, Abcam), rabbit polyclonal anti-phosphorylated GATA4 (anti-pGATA4) (1:500, phosho-S105, ab92585, Abcam), goat polyclonal anti-GATA4 (1:50, sc-1237, Santa Cruz Biotechnology), mouse monoclonal anti-smooth muscle actin (1:100, IS61130, DAKO, Glostrup, Denmark), rabbit polyclonal anti-phosphorylated STAT3 (p-STAT3) (1:100, #9131, Cell Signaling Technologies, Danvers, MA, USA), rabbit polyclonal phospho-p44/42 MAPK (p-ERK) (1:50, #9101, Cell Signaling Technologies, Danvers, MA, USA), rabbit polyclonal Akt1/2/3 (Ser 473)-R (p-Akt) (1:50, sc-7985-R, Santa Cruz Biotechnology), rabbit polyclonal anti-vimentin (1:20, GP59, Progen, Heidelberg, Germany), rat monoclonal anti-CD31 (1:100, 14-0311-81, eBioscience, San Diego, CA, USA), rat monoclonal anti-mouse Ly-6A/E (Sca-1) (1:100, 14-5981-81, eBioscience), rabbit polyclonal connexin 40 (1:100, AB1726, Merck Millipore, Darmstadt, Germany), and rabbit polyclonal Ki67 antibody (1:100, ab15580, Abcam). Alexa Fluor488 (A21202 and A21206, Invitrogen, Carlsbad, CA), Cy3- and Cy5-conjugated secondary antibodies (711-165-152, 712-165-150, and 715-175-150, Jackson ImmunoResearch Laboratory, West Grove, PA, USA) were used.

### Image analysis

Images of samples were collected by laser confocal microscopy (Radiance 2000; Bio-Rad Laboratories, Hercules, CA, and TCS-SP5 Ver 2.0; Leica Microsystems, Solms, Germany) or fluorescence microscopy (Carl Zeiss MicroImaging, Inc., Oberkochen, Germany) with a CCD camera (Axiocam; Carl Zeiss MicroImaging, Inc.) Cardiomyocytes were defined as cells with clear sarcomeres delineated by laminin. The MI remote area was defined as the area more than one high-power field away from the scar tissue. The cross-sectional areas of the cardiomyocytes were analyzed using Adobe Photoshop software (AdobeSystems, San Jose, CA, USA). The frequency of X-gal-negative cardiomyocytes and nuclear Nkx2.5-positive cardiac progenitors are represented as the numbers of these cells divided by the evaluated areas.

### Echocardiography

Transthoracic echocardiography was performed with an Agilent Sonos 4500 (Agilent Technologies Co., Santa Clara, CA, USA) with an 11-MHz imaging transducer. M-mode images of the LV were recorded.

### RNA isolation and quantitative real-time polymerase chain reaction (qRT-PCR)

Total RNA was extracted from cardiac tissue and CSP cells using RNA-bee reagent (Cs-501B, Tel-Test Inc., Friendswood, TX, USA). NIH 3T3 cells were used as the positive control for LIFR [23]. qRT-PCR was performed using a LightCycler (Roche, Basel, Switzerland) with the Taqman Universal Probe Library and the LightCycler Master (Roche), according to the manufacturer’s instructions. The primers were as follows: GAPDH reverse, 5´-AAAAAAACCCCCCCCC-3´, and forward, 5´-CCCCCTTTTTTTTTTTTTTT-3´; β-galactosidase reverse, 5´-GGCGATTAAGTTGGGTAACG-3´ and forward, 5´-CACTGGCCGTCGTTTTACA-3´; and LIFR reverse, 5´-AGCAACATGGTAAGTTGAATCCT-3´ and forward, 5´-GCTAATTCCAAGAAAGAAGTGAGG-3´.

### LIF concentration assay

The plasma LIF concentration was measured using an enzyme-linked immunosorbent assay kit (Quantikine M, MLF00, R&D Systems, Minneapolis, MN, USA; LIF/HILDA EASIA, KAC1351, Life Technologies, Carlsbad, CA, USA) according to the manufacturer’s instructions.

### BrdU labeling

To identify cells that participate in the cell cycle, injections of BrdU (B5002, Life Technologies, 25 mg/kg, ip) were administered every 12 h for 9 days from the time of surgery. To identify LRCs, BrdU (50 mg/kg ip) was injected into pregnant females every 24 h, for a total of two injections, 2–4 days prior to parturition. The newborn mice were healthy, and mice with the CreLacZ phenotype were used in the analysis.

### EdU labeling

To detect CSPs that participate in the cell cycle, 5-ethynyl-2′-deoxyurinide (EdU, A10044, Life Technologies, 25 mg/kg, ip) was administered to PBS- and LIF-treated mice at 5 and 6 days after MI, and the CSPs were isolated on day 7 as previously described. The CSPs were fixed, and EdU staining was performed according to the manufacturer’s instructions, followed by MDR1 and DAPI staining.

### Flow cytometric analysis of phosphorylated STAT3

Freshly isolated SP cells were fixed in a single step using BD™ Phosflow Lyse/Fix buffer for 10 min at 37°C. The cells were subsequently permeabilized in BD™ Phosflow Perm Buffer III for 30 min on ice, washed twice in BD Pharmingen™ Stain Buffer and stained with PE mouse anti-STAT3 (pY705) antibody for 30 min at room temperature. This staining was performed according to the manufacturer’s instructions using BD™ Phosflow reagents (612569, BD Biosciences, Franklin Lakes, NJ, USA). The samples were analyzed using a BD FACSCalibur™ instrument.

### Data analysis

The results are presented as the mean ± SEM. Multiple group comparisons were performed using the Kruskal–Wallis test or one-way analysis of variance, followed by Bonferroni’s procedure to compare means. Comparisons between two groups were conducted using a two-tailed Student’s t-test, two-tailed Welch’s t-test, or Mann–Whitney U-test. P < 0.05 was considered statistically significant.

## Results

### Efficiency and specificity of tamoxifen-induced β-galactosidase expression in cardiomyocytes of CreLacZ mice

The CreLacZ (Fig 1A) mice were fertile with normal cardiac histology (S1 Fig.) and echocardiographic function (S1 Table). Following tamoxifen administration, the cardiomyocytes, which were characterized by sarcomere formation and surrounding laminin, exhibited X-gal-positive staining in the LV (Fig 1B). β-galactosidase expression in the cardiomyocytes was confirmed by immunofluorescence using an anti-β-galactosidase antibody (S1 Fig). A limited number of X-gal-negative cardiomyocytes was identified at the crest of the muscular ventricular septum and the endocardial surface of the LV immediately after completion of tamoxifen treatment. These cells were from the cardiac conduction system, including the atrioventricular node, bundle of His, and Purkinje fibers, as judged by their location and connexin 40 expression (S1 Fig). Therefore, we excluded the cardiomyocytes within two layers from the endocardium and the basal ventricular septum from the regions of interest in subsequent analyses. Immediately after completion of tamoxifen treatment, 99.9 ± 0.1% of the working cardiomyocytes (Fig 1C) in a region of interest were labeled. Non-cardiomyocytes, such as smooth muscle cells, endothelial cells, and interstitial fibroblasts, indicated virtually no β-galactosidase activity following tamoxifen treatment (S2 Fig). X-gal staining was positive in 0.8 ± 0.5% of the smooth muscle actin-positive, 0.8 ± 0.4% of the vimentin-positive, and 0.7 ± 0.3% of the CD31-positive cells. Flow cytometric analysis indicated that 82.4% of the cells isolated from a CreLacZ mouse LV were FDG-positive and predominately comprised α-MHC-positive and X-gal-positive pre-existing cardiomyocytes (S2 Fig). FDG-negative cells consisted of a substantial number of α-MHC and X-gal double-positive cardiomyocytes. All α-MHC-negative cells in the FDG-negative fraction were X-gal-negative. In this experiment, the CreLacZ mice were sacrificed immediately after completion of tamoxifen treatment; thus, de novo cardiomyocyte formation should be rare. Therefore, the sorted FDG-negative fraction contained a substantial number of pre-existing cardiomyocytes. These findings suggest that X-gal staining was sufficiently sensitive and specific to measure X-gal-negative cardiomyocytes (S2 Fig). Nearly 100% of the working cardiomyocytes were irreversibly labeled with β-galactosidase in the CreLacZ mice, which enabled us to determine the emergence of β-galactosidase-negative cardiomyocytes as a marker of de novo cardiomyogenesis, a process that involved non-cardiomyocytes but not pre-existing mature cardiomyocytes.

### Increase in X-gal-negative newly formed cardiomyocytes after cardiac injury

First, we determined whether the number of newly formed cardiomyocytes increased with normal aging. The assessment of β-galactosidase expression at different time points over a 1-year period in CreLacZ mice treated with tamoxifen indicated that the number of X-gal-negative cardiomyocytes remained essentially unchanged (1 day: 0.29 ± 0.06 cells/mm^2^; 6 months: 0.44 ± 0.13 cells/mm^2^; 12 months: 0.56 ± 0.17/mm^2^; Fig 1D). This finding suggested that the involvement of non-cardiomyocytes in the generation of new cardiomyocytes is rare during normal aging under physiological conditions.

**Fig 2:**
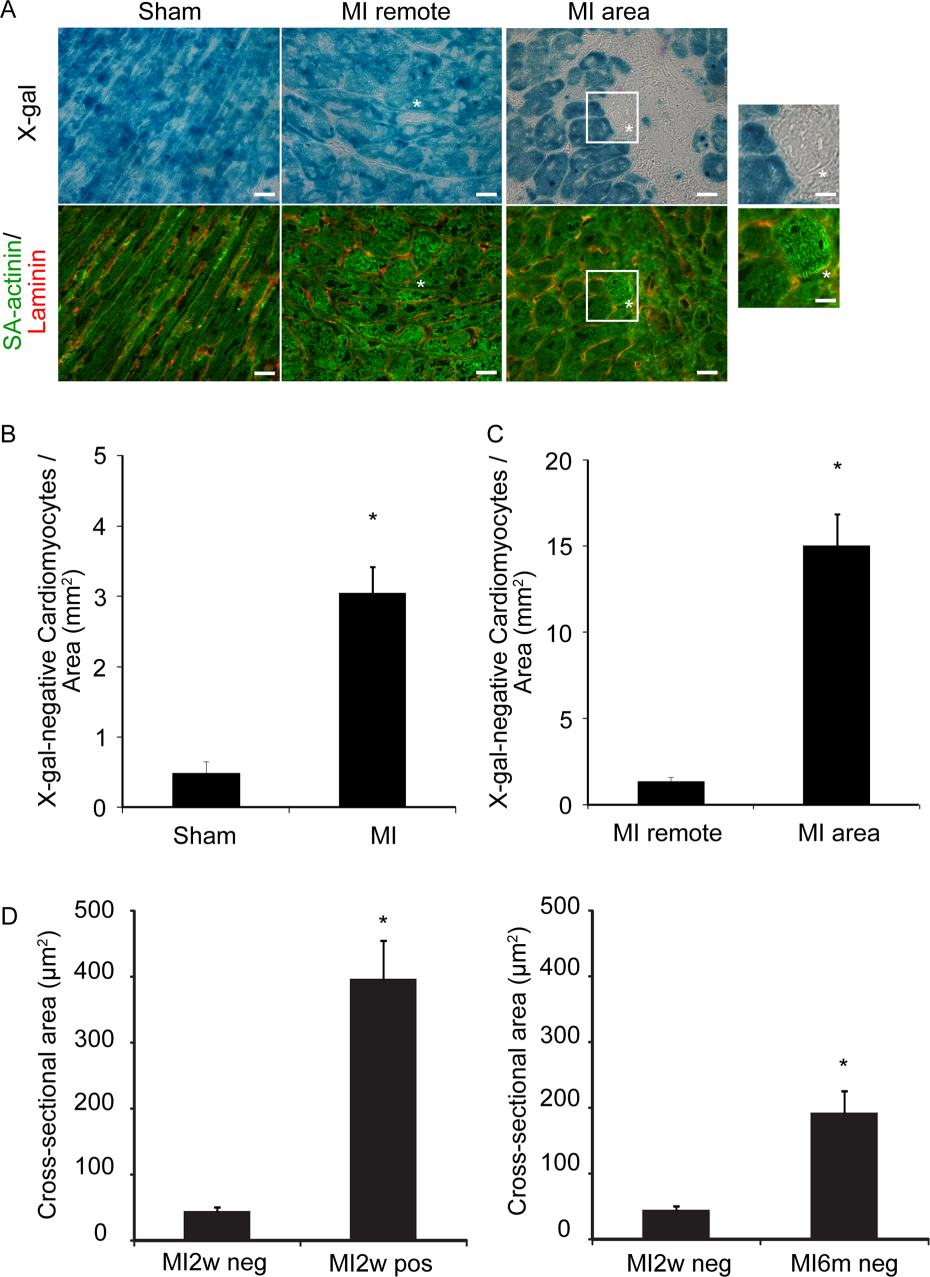
X-gal-negative Newly Formed Cardiomyocytes Increase Following Cardiac Injury. (A) X-gal staining and immunofluorescent images of sections comparing sham and myocardial infarction (MI) CreLacZ mice: left panel, sham; middle panel, MI remote area; and right panel, MI area. The upper panels show X-gal-stained images. The bottom panels show the corresponding immunofluorescent images (SA-actinin, green; laminin, red). Asterisks show X-gal-negative cardiomyocytes. Scale bar, 20 μm. The X-gal-negative cardiomyocytes are further enlarged in the inset. Scale bar, 10 μm. (B) Number of X-gal-negative cardiomyocytes per area 3 months after MI: sham (n = 5) and MI (n = 5). *p < 0.05. (C) Comparison of the number of X-gal-negative cardiomyocytes in the MI remote and MI area 3 months after MI (n = 5 per area). *p < 0.05. (D) Left: comparison of the cross-sectional area between X-gal-negative (MI2w neg) and -positive (MI2w pos) cardiomyocytes at 2 weeks after MI. Right: comparison of the cross-sectional area of X-gal-negative cardiomyocytes at 2 weeks (MI2w neg) and 6 months (MI6m neg) after MI. n = 50–66 cardiomyocytes pooled from two MI mice per group. *p < 0.05. The Mann–Whitney U-test was used for statistical analysis.

We subsequently examined whether cardiac injury increases the number of newly formed cardiomyocytes. Following tamoxifen administration, the CreLacZ mice were randomly divided into sham and MI groups (n = 5 per group). As shown in Fig 2A, X-gal-negative cardiomyocytes were present in the MI border area at 3 months after infarction. These cardiomyocytes were small; however, they exhibited SA-actinin-positive fine sarcomeres and were delineated by laminin. The number of X-gal-negative cardiomyocytes in the MI group was significantly increased compared with the sham control group at 3 months after infarction (MI: 3.04 ± 0.38 cells/mm^2^; sham: 0.47 ± 0.16 cells/mm^2^; p < 0.05; Fig 2B). The X-gal-negative cardiomyocytes were predominantly present in the MI area, including the border and infarct area (remote area: 1.33 ± 0.19 cells/mm^2^; MI area: 15.02 ± 1.78 cells/mm^2^; p < 0.05; Fig 2C). These findings suggested that MI stimulates differentiation of non-cardiomyocytes into cardiomyocytes. The cross-sectional area of X-gal-negative cardiomyocytes (45.50 ± 6.14 μm^2^) was significantly smaller than that of X-gal-positive cardiomyocytes (397.48 ± 58.50 μm^2^) at 2 weeks after MI (p < 0.05, Fig 2D). The size of the X-gal-negative cardiomyocytes significantly increased at 6 months after MI (192.99 ± 32.88 μm^2^) compared with 2 weeks after MI (p < 0.05, Fig 2D). These data are consistent with the notion that X-gal-negative cardiomyocytes originate from premature cells and increase in size over several months.

### X-gal-negative newly formed cardiomyocytes originate from label-retaining CSCs or CPCs

**Fig 3:**
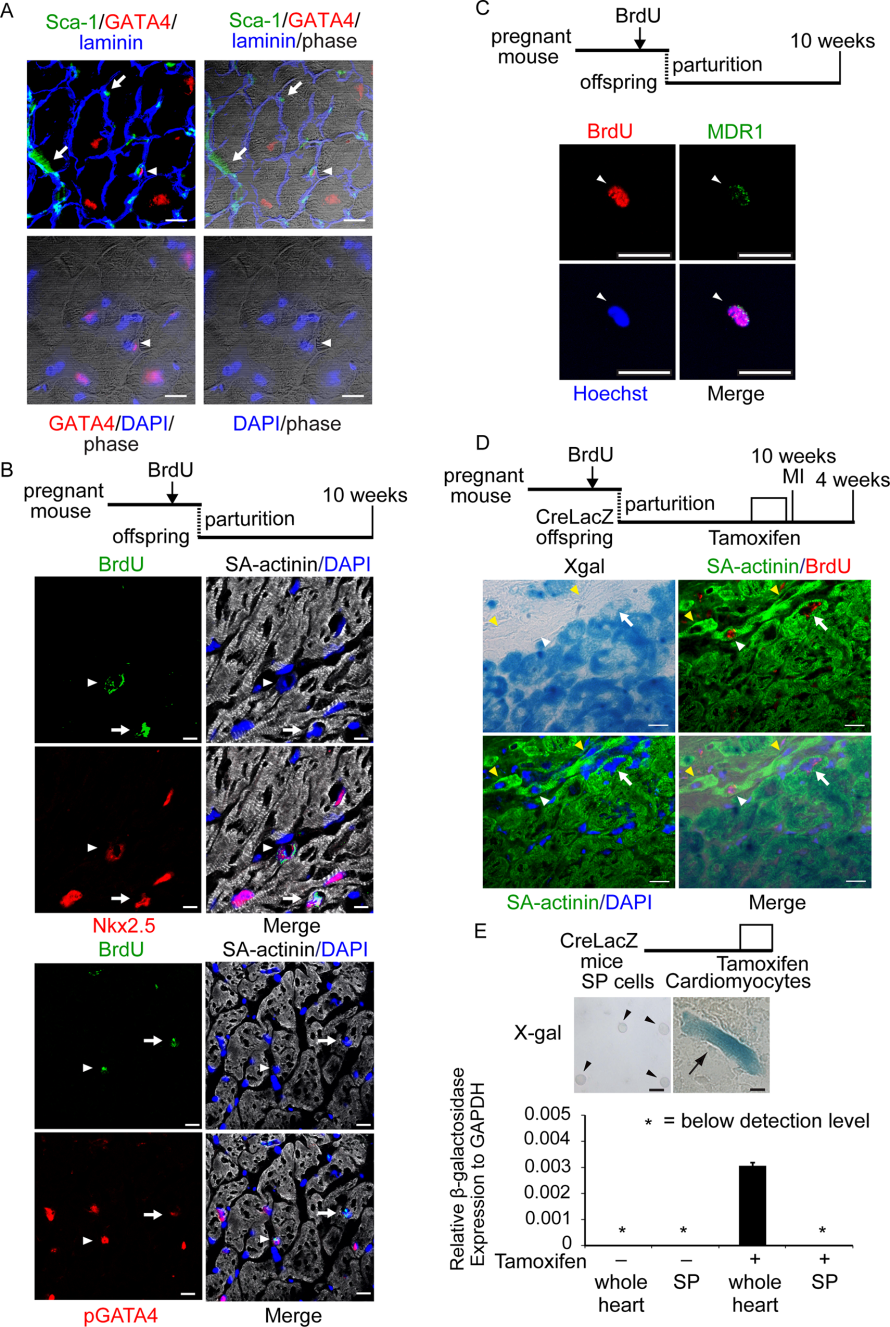
X-gal-negative Newly Formed Cardiomyocytes Originate from LRCs, Which Consisted of CSCs or CPCs. (A) A CPC co-expressing Sca-1 and GATA4. In the two left panels, the arrowhead indicates a Sca-1 (green) and GATA4 (red) double-positive CPC surrounded by a basement membrane of cardiomyocytes (laminin in blue). The GATA4 signal co-localized with DAPI nuclear staining (blue) in the two right panels. Arrows indicate Sca-1-positive capillaries. Scale bar, 10 μm. (B) Partial overlap of LRCs and CPCs. The hearts of 10-week-old mice that were administered BrdU during the fetal period were immunohistochemically examined. Some anti-sarcomeric α-actinin (SA-actinin)-negative and BrdU-positive LRCs were Nkx2.5-positive (arrowhead, upper panels) or pGATA4-positive (arrowhead, lower panels). Some Nkx2.5-positive (arrow, upper panels) or pGATA4-positive (arrow, lower panels) cardiomyocytes retained BrdU. Nuclei were stained with DAPI. Scale bar, 10 μm. (C) Partial overlap of LRCs and CSCs. Cardiac side population cells (CSPs) were isolated from the hearts of 10-week-old mice that had been administered BrdU during the fetal period. BrdU-positive and multi-drug-resistant protein 1(MDR1)-positive CSPs were identified (arrowheads). Scale bar, 10 μm. (D) X-gal-negative newly formed cardiomyocytes originate from LRCs. A white arrowhead indicates SA-actinin-positive and X-gal-negative LRC-derived newly formed cardiomyocytes. An arrow indicates SA-actinin- and X-gal-positive pre-existing cardiomyocytes. Scale bar, 10 μm. Note that the nuclei of both cell types retained BrdU because of their quiescence after birth. Two yellow arrowheads indicate SA-actinin-positive and X-gal-negative cardiomyocytes, the ancestral CSCs or CPCs of which circumvented the BrdU labeling because of their quiescence. (E) Cre-mediated recombination does not occur in CSPs after tamoxifen treatment. X-gal staining of the cardiomyocyte fraction (right) and CSP cell fraction (left) from a tamoxifen-treated mouse. Arrowheads indicate X-gal-negative CSPs. An arrow indicates X-gal-positive cardiomyocytes. β-galactosidase mRNA expression in cardiomyocyte suspension and sorted CSP fraction from tamoxifen-treated and -untreated mice. Scale bar, 10 μm.

We examined whether X-gal-negative cardiomyocytes originate from CSCs or CPCs, which divide infrequently in an undamaged heart but have the ability to proliferate in response to injury [24]. We repeatedly injected pregnant mice with BrdU to label the dividing fetal heart cells at a time of rapid tissue expansion and identify CSCs or CPCs that do not subsequently divide and thus retain the label into adulthood (label-retaining cells, LRCs) [25]. Various types of CPCs that express stem cell-associated markers, such as c-kit, MDR1, or Sca-1, share several features, including an ability to differentiate into cardiovascular lineages, a lack of contractile proteins, and expression of the cardiac transcription factors Nkx2.5 and GATA4 [26]. A typical example of a CPC is shown in Fig 3A (arrowhead). The CPC was localized in the interstitial space, which was surrounded by the basement membrane of the cardiomyocytes (laminin) expressing cell surface Sca-1 and nuclear GATA4. Sca-1 was also expressed in GATA4-negative capillary endothelial cells (arrows). We determined whether LRCs share features of CPCs based on the nuclear localization of Nkx2.5 and pGATA4. At 10 weeks of age, some BrdU-positive LRCs were Nkx2.5-positive (Fig 3B, upper panels, arrowhead) or pGATA4-positive (Fig 3B, lower panels, arrowhead), and the fluorescent signals overlapped with the DAPI-stained nuclei (Fig 3B). Quantitatively, 10.0% and 19.4% of the LRCs were pGATA4-positive and Nkx-2.5-positive CPCs, respectively. These cells were located between cardiomyocytes and were SA-actinin-negative. Therefore, these BrdU-positive and Nkx2.5- or pGATA4-positive cells were CPCs, which were successfully labeled as LRCs. Some Nkx2.5-positive (Fig 3B, upper panels, arrows) or pGATA4-positive (Fig 3B, lower panels, arrows) cardiomyocytes retained BrdU because proliferative cardiomyocytes in the fetal mouse heart withdraw from the cell cycle during the first few days after birth [27]. We previously reported that CSPs are CSCs, which are capable of differentiating into cardiomyocytes both in vivo and in vitro [21]. The molecular determinant of the SP phenotype in the adult heart is MDR1, which efficiently effluxes Hoechst 33342 and enables CSPs to be identified by flow cytometry [28]. When CSPs were isolated from mice exposed to BrdU during the fetal period, 14.8 ± 1.7% of the MDR1-positive CSPs were BrdU-positive (S3 Fig and Fig 3C); thus, some CSCs were labeled as LRCs.

Subsequently, we assessed whether new X-gal-negative cardiomyocytes were LRCs. Using a previously described protocol, we injected tamoxifen into the mice that had been exposed to BrdU during the fetal period and subsequently induced MI. Four weeks after infarction, X-gal-negative BrdU-positive cardiomyocytes were identified in the MI area (Fig 3D, white arrowhead). When we examined the X-gal-negative cardiomyocytes in which the nuclei were sectioned, 10.5 ± 4.3% of the X-gal-negative cardiomyocytes were BrdU-positive (mean of three heart sections each from five MI mice). These X-gal-negative and BrdU-positive cardiomyocytes were the descendants of CPCs or CSCs that had actively proliferated 2–4 days prior to parturition and subsequently become quiescent after birth. The X-gal-positive pre-existing cardiomyocytes also retained BrdU, indicating that the cardiomyocytes divided during the fetal stage or label-retaining CSCs differentiated into cardiomyocytes prior to tamoxifen administration (Fig 3D, arrow). In total, 98.5% of the CSPs isolated from the CreLacZ mice immediately after tamoxifen treatment were X-gal-negative (Fig 3E, left panel, arrowheads). The non-CSPs contained 83.3% X-gal-positive cells, which were thought to be cardiomyocytes (Fig 3E, right panel, arrow). qRT-PCR analysis indicated that CSPs from a tamoxifen-treated mouse did not express β-galactosidase mRNA, but expression was detected in whole heart tissue lysates from a tamoxifen-treated mouse (Fig 3E). These findings suggested that some X-gal-negative, newly formed cardiomyocytes differentiate from non-cardiac LRCs, which are characterized by a slow-cycling stem cell property and are present as CPCs or CSCs in adult mice.

### LIF increases the number of newly formed X-gal-negative cardiomyocytes

We have previously reported that LIF administration attenuates the extent of the infarct and fibrosis after MI [9]. Therefore, we investigated whether LIF stimulates the proliferation and/or differentiation of dormant CSCs. Injection of LIF cDNA into the thigh muscle of mice immediately after MI maintained the increased blood LIF levels at 1,603 pg/mL on day 3, 5,500 pg/mL at 1 week, and 500 pg/mL at 2 weeks after injection. Injection of PBS immediately after MI resulted in no detectable increase in blood LIF levels at these time-points. In the LIF-treated group, the fibrosis level was significantly decreased compared with the vehicle-treated group (S4 Fig). Fractional shortening significantly improved in the LIF-treated group compared with the control group at 1 month after MI (S4 Fig and S2 Table).

**Fig 4:**
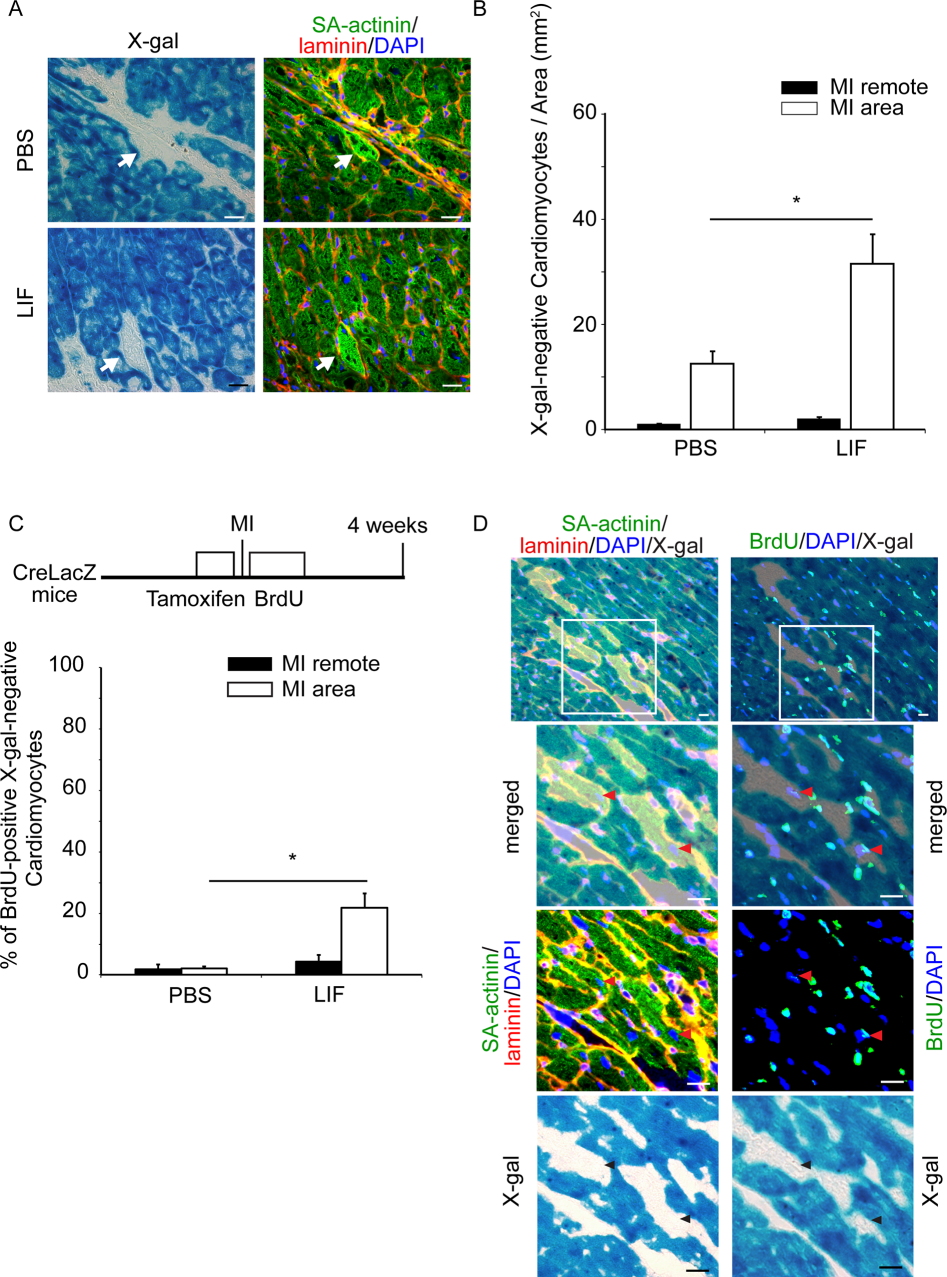
LIF Increases the Number of X-gal-negative Newly Formed Cardiomyocytes and the Frequency of BrdU Incorporation. (A) Representative images of X-gal-negative cardiomyocytes in PBS- and LIF-treated CreLacZ mice. X-gal staining (left) and immunofluorescence images (right; SA-actinin, green; laminin, red; nuclei were stained with DAPI, blue) are shown. Arrows indicate X-gal-negative cardiomyocytes. Scale bar, 20 μm. (B) Number of X-gal-negative cardiomyocytes in the MI remote area (closed bar) and the MI area (open bar) in the PBS- and LIF-treated mice after MI. Asterisks indicate significant differences between two groups. *p < 0.05. (C) Frequencies of BrdU-positive X-gal-negative cardiomyocytes among all X-gal-negative cardiomyocytes in the MI remote area (closed bar) and the MI area (open bar) in the PBS- and LIF-treated mice after MI. Asterisks indicate significant differences between two groups. *p < 0.05. Three pairs of adjacent heart sections were examined per mouse. Data indicate the mean of five mice. (D) Representative images of a pair of adjacent sections. Left panels were stained with SA-actinin (green), laminin (red), DAPI (blue), and X-gal. Right panels were stained with BrdU (green), DAPI (blue) and X-gal. Top panels represent a group of X-gal-negative cardiomyocytes in the MI area of a LIF-treated mouse. The enlarged images of a region of interest (white square) are shown. Two X-gal-negative cardiomyocytes (arrowheads in the left panels) and the corresponding BrdU-positive nuclei (arrowheads in the right panels) are shown. Scale bar, 20 μm.

Subsequently, we compared the number of X-gal-negative cardiomyocytes in the LIF-treated CreLacZ mice at 28 days after LIF plasmid injection with the PBS-treated CreLacZ mice. In the MI area, the LIF-injected mice had 31.41 ± 5.83 X-gal-negative cardiomyocytes/mm^2^ (n = 6), whereas the PBS-injected mice had 12.34 ± 2.56 X-gal-negative cardiomyocytes/mm^2^ (n = 5; p < 0.05; Fig 4A and 4B). When the total number of X-gal-negative cardiomyocytes in the MI area was estimated based on a morphometric analysis, the LIF-injected mice regenerated 1.96 ± 3.33 × 10^4^ cardiomyocytes (n = 6), and the PBS-injected mice regenerated 0.93 ± 0.17 × 10^4^ cardiomyocytes (n = 5). In the MI remote area, the difference between the two groups was not significant: the LIF-injected mice had 0.85 ± 0.17 X-gal-negative cardiomyocytes/mm^2^ (n = 6), whereas the control mice had 1.80 ± 0.51 cells/mm^2^ (n = 5). Thus, the increase in X-gal-negative cells was mainly attributed to the increase in the MI area. To elucidate whether LIF stimulated the proliferation of developing cardiomyocytes, BrdU was injected into PBS- or LIF-treated CreLacZ mice every 12 h for 9 days after MI, and the frequency of BrdU incorporation in the X-gal-negative cardiomyocytes was measured at 4 weeks. A higher percentage of BrdU-positive and X-gal-negative cardiomyocytes was identified in the LIF-treated mice than in PBS-treated mice in the MI area (1.9 ± 0.8% in PBS-treated mice, 21.8 ± 4.5% in LIF-treated mice, p < 0.05) but not in the MI remote area (1.9 ± 1.3% in PBS-treated mice, 4.3 ± 2.2% in LIF-treated mice; Fig 4C). Immunohistochemical images of a pair of adjacent frozen sections obtained from the LIF-treated mice indicated that two X-gal-negative and SA-actinin-positive cardiomyocytes delineated by laminin (arrowheads in left panels) possessed BrdU-positive nuclei (arrowheads in right panels) (Fig 4D).

**Fig 5:**
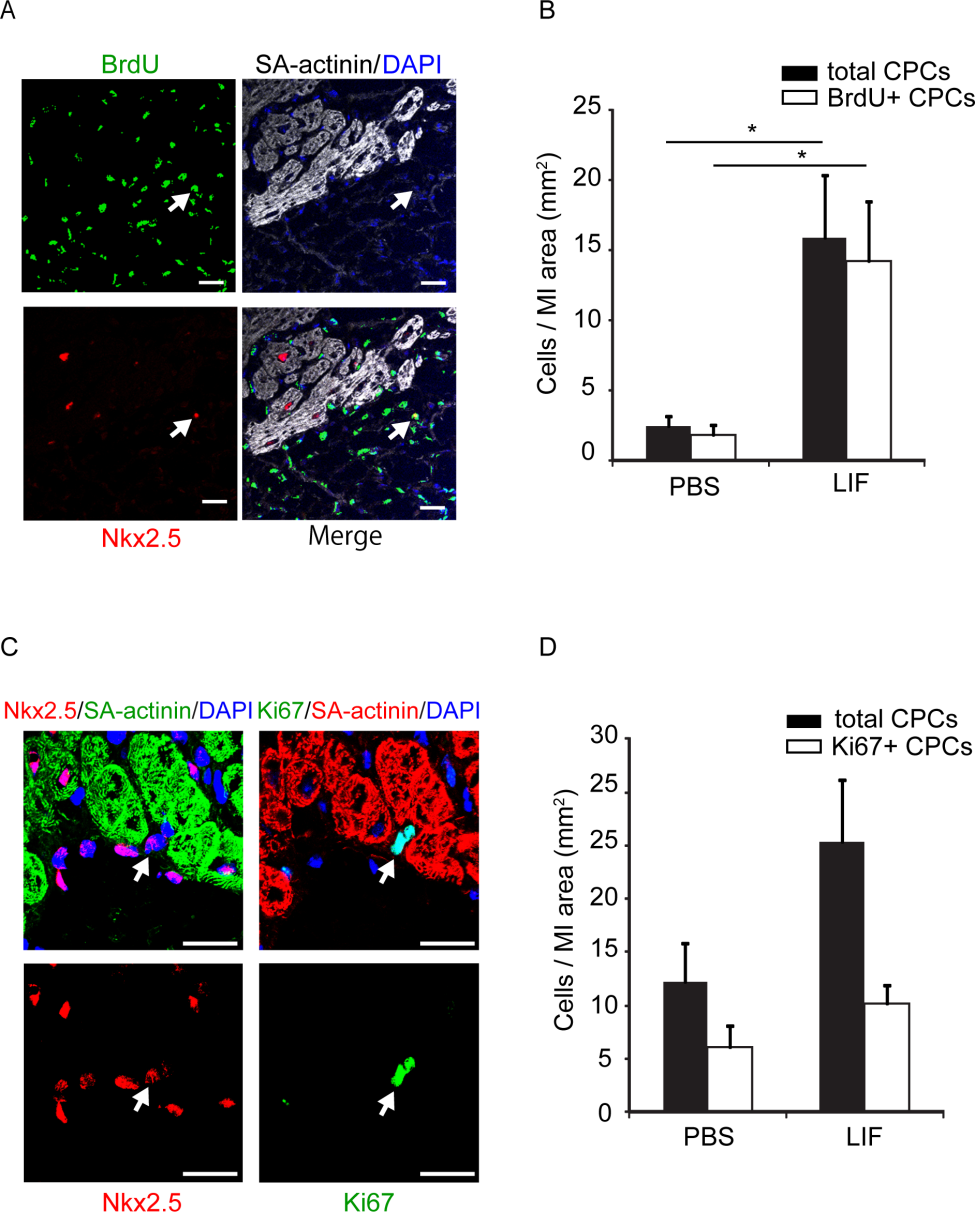
LIF Increases the Number of Proliferative CPCs. (A) Proliferative CPCs in the border of an MI heart. An arrow indicates a BrdU-positive (green), Nkx2.5-positive (red), and SA-actinin-negative (white) CPC. Nuclei were stained with DAPI (blue). Scale bar, 20 μm. (B) Number of total CPCs (closed bar) and BrdU-positive CPCs (open bar) in the PBS- and LIF-treated mice after MI. Asterisks indicate significant differences between two groups. *p < 0.05. (C) LIF-injected or PBS-injected mice were sacrificed at 1 week after MI. An arrow in the left two panels indicates the Nkx2.5-positive (red) SA-actinin-negative (green) cells. The serial adjacent sections in the right two panels indicate that the same cell (arrow) was also Ki67-positive (green) and SA-actinin-negative (red). Nuclei were stained with DAPI (blue). Scale bar, 10 μm. (D) Number of Ki67 and Nkx2.5 double-positive cells per area in the PBS- and LIF-treated mice after MI.

**Fig 6:**
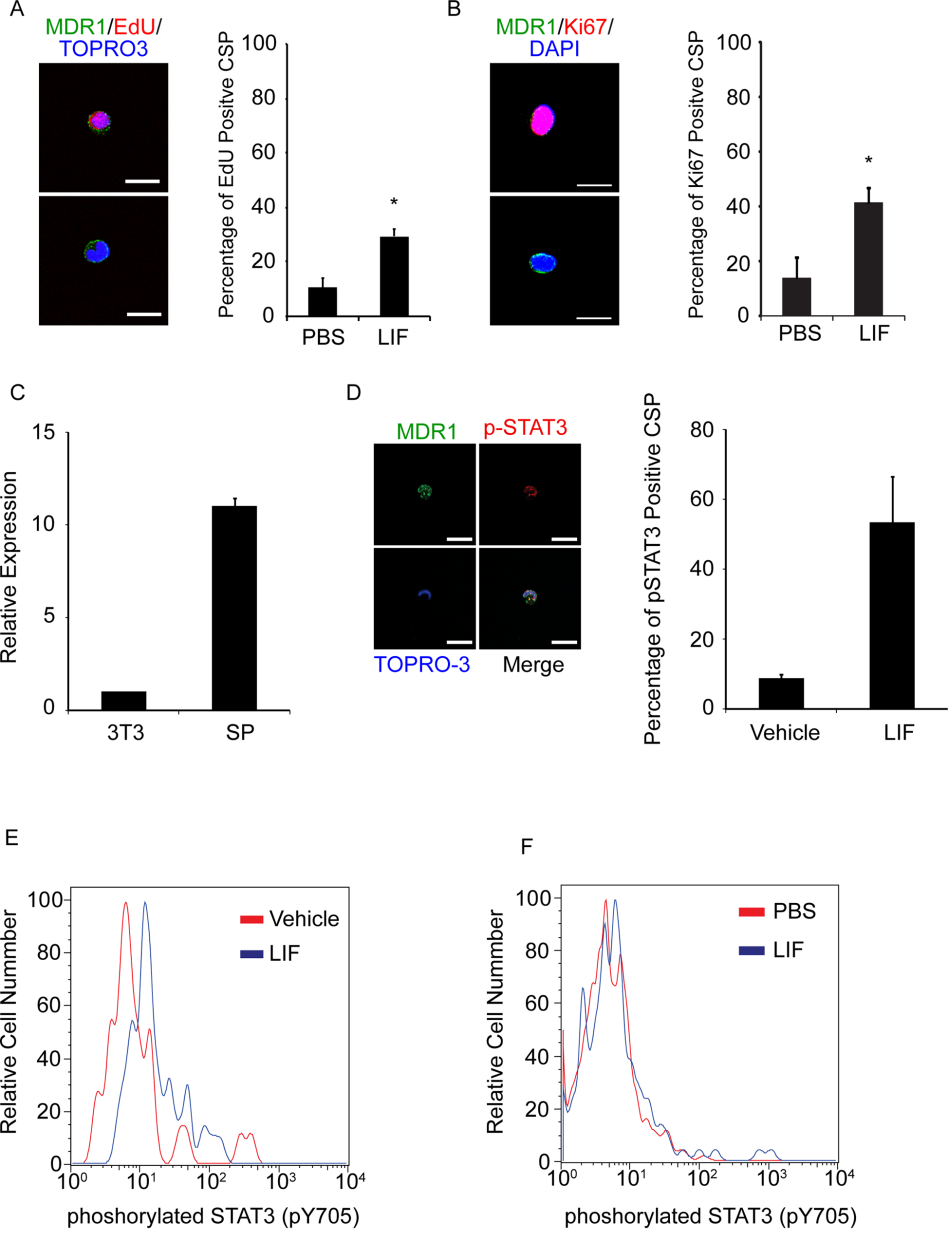
LIF Activates the JAK-STAT Pathway and Induces Cell Cycle Re-entry in Cardiac CSPs. (A) Representative images of CSPs stained with antibodies to MDR1 (green), EdU (red), and TO-PRO-3 (blue). EdU-positive (upper panel) and -negative (lower panel) CSPs are shown. Scale bar, 10 μm. The bar graph indicates the percentage of EdU-positive CSPs among all CSPs isolated from the PBS- and LIF-treated mice at 1 week after MI. *p < 0.05. (B) Representative images of CSPs stained with antibodies for MDR1 (green), Ki67 (red), and DAPI (blue). Ki67-positive (upper panel) and -negative (lower panel) CSPs are shown. Scale bar, 10 μm. The bar graph indicates the percentage of Ki67-positive CSPs among all CSPs isolated from the PBS- and LIF-treated mice at 1 week after MI. *p < 0.05. (C) LIFR mRNA expression in CSPs compared with NIH 3T3 cells. (D) LIF promotes nuclear p-STAT3 accumulation in isolated CSPs. Representative images of p-STAT3-positive CSPs stained with antibodies to MDR1 (green), p-STAT3 (red), and TO-PRO-3 (blue). Scale bar, 20 μm. The bar graph indicates the percentage of p-STAT3-positive CSPs among all CSPs. (E) Number of p-STAT3-positive CSPs following exposure to vehicle (red line) and LIF (blue line) using flow cytometry. Data represent three independent experiments. (F) Number of p-STAT3-positive CSPs isolated from control mice (red line) and LIF-treated mice (blue line) using flow cytometry at one week after MI. Data represent three independent experiments.

We subsequently investigated whether LIF administration promoted the proliferation of undifferentiated cardiomyocytes such as CPCs and CSCs. As shown in Fig 3D, some X-gal-negative cardiomyocytes differentiated from LRCs, which had CPC or CSC characteristics. PBS- or LIF-treated mice received a BrdU injection every 12 h for 9 days after MI and were sacrificed at 2 weeks; SA-actinin-negative, Nkx2.5-positive, and BrdU-positive proliferative CPCs were identified in the MI area (Fig 5A). The numbers of both SA-actinin-negative and Nkx2.5-positive total CPCs and BrdU-positive proliferative CPCs were significantly increased in the LIF-treated mice compared with the PBS-treated mice (total CPCs: 2.41 ± 0.76 cells/mm^2^ in PBS-treated mice, 15.87 ± 0.35 cells/mm^2^ in LIF-treated mice, p < 0.05; BrdU-positive proliferative CPCs: 1.86 ± 0.70 cells/mm^2^ in PBS-treated mice, 14.19 ± 4.35 cells/mm^2^ in LIF-treated mice, p < 0.05; n = 6 mice per group, one section was examined per mouse; Fig 5B). In general, the sections from the LIF-treated mice exhibited more Ki67 and Nkx2.5 double-positive CPCs; however, the difference was not significant (total CPCs: 12.21 ± 3.56 cells/mm^2^ in PBS-treated mice, 25.31 ± 5.91 cells/mm^2^ in LIF-treated mice; Ki67-positive proliferative CPCs: 6.04 ± 2.13 cells/mm^2^ in PBS-treated mice, 10.22 ± 1.62 cells/mm^2^ in LIF-treated mice; n = 3 mice per group, one section was examined per mouse. Fig 5C and 5D). When EdU was administered to the PBS- and LIF-treated mice at 5 and 6 days after MI and the CSP EdU-positive rate was measured on day 7, there were significantly more EdU-positive proliferative CSP cells in the LIF-treated mice (PBS-treated mice: 10.6 ± 3.7%; LIF-treated mice: 29.4 ± 2.7%; p < 0.05; n = 6 and 4, respectively; Fig 6A). When CSPs were isolated and immunostained for Ki67 at 1 week after MI, there were significantly more Ki67-positive proliferative CSPs in the LIF-treated mice (PBS-treated mice: 14.0 ± 7.3%; LIF-treated mice: 41.4 ± 5.4%; p < 0.05; n = 3 per group; Fig 6B).

To determine whether LIF activates the downstream signaling pathway in CSPs, we added LIF (2000 U/mL) to freshly isolated SP cells. CSPs express substantial amounts of LIFR mRNA compared with NIH 3T3 cells (Fig 6C). Five minutes of LIF exposure led to 53.3 ± 13.3% p-STAT3-positive CSPs, whereas only 8.4% ± 1.5% of CSPs were p-STAT3-positive in the control group (n = 2 per group; Fig 6D). Flow cytometric analysis also indicated that the p-STAT3 levels in the CSPs increased after 10 min of LIF exposure in vitro (Fig 6E). The p-STAT3 levels increased in the CSPs that were isolated from the LIF-treated mice at 1 week after MI (Fig 6F). PI3K-AKT and MEK-ERK pathway activation was also demonstrated following LIF treatment (S5 Fig). Twenty minutes of LIF exposure led to 90.8 ± 1.9% p-Akt-positive CSPs, whereas 51.4 ± 0.9% of the CSPs were p-Akt-positive in the control group (n = 2 per group; S5 Fig). LIF exposure also led to 59.4 ± 5.8% p-ERK-positive CSPs, whereas 17.5 ± 2.6% of the CSPs were p-ERK-positive in the control group (n = 2 per group; S5 Fig). When CSPs were isolated from the LIF-treated and PBS-treated mice 1 week after MI, the percentages of p-Akt- and p-ERK-positive cells were significantly increased in LIF-treated mice (89.7 ± 0.4% of p-Akt-positive CSPs in LIF-treated mice and 63.4 ± 4.3% in PBS-treated mice, p < 0.05, n = 3 per group; 62.9 ± 7.5% p-ERK-positive CSPs in LIF-treated mice and 37.6 ± 7.5% in PBS-treated mice, p < 0.05, n = 3 per group; S5 Fig).

### BM-derived cells cannot account for the increase in X-gal-negative cardiomyocytes

**Fig 7:**
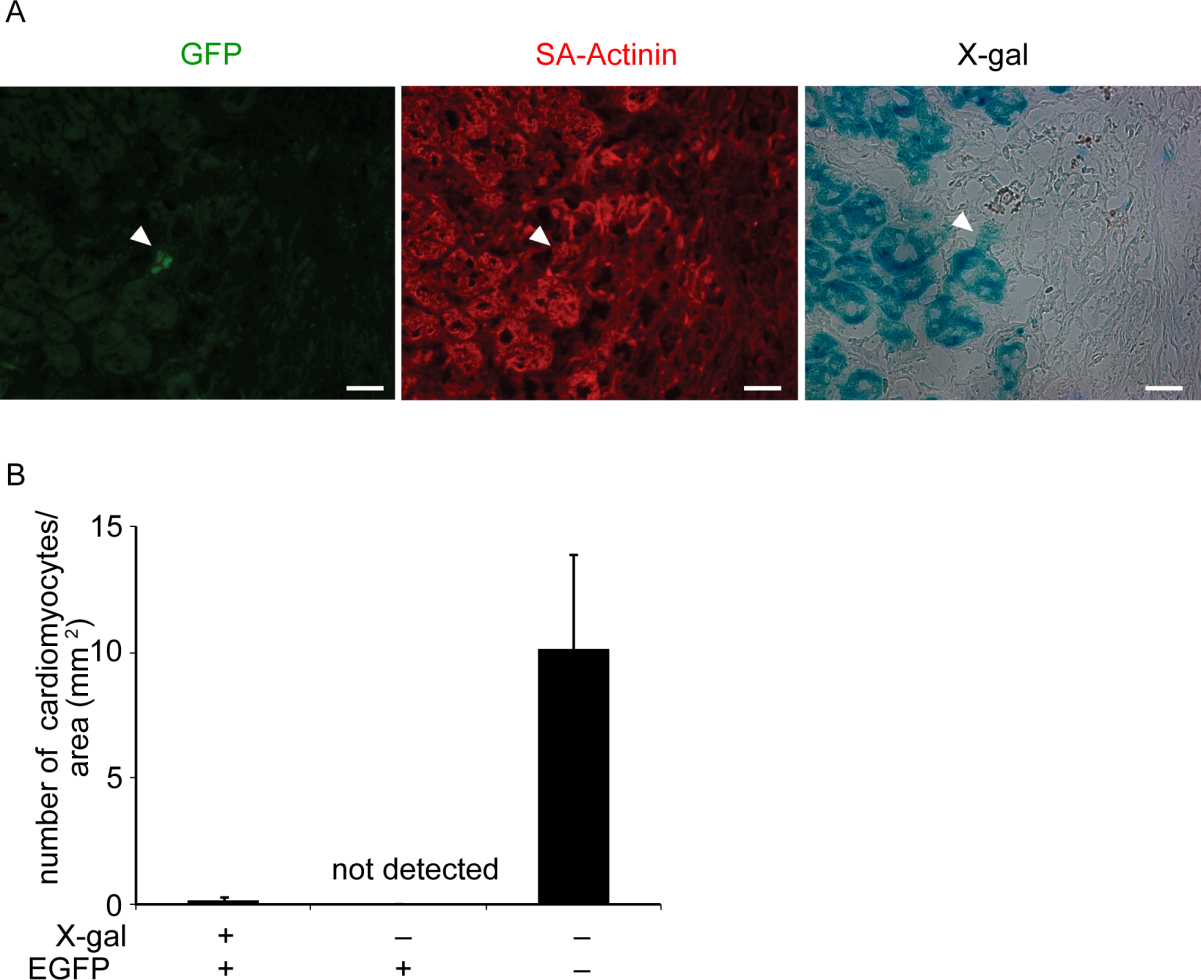
Bone Marrow-derived Cells Do not Account for the Increase in X-gal-negative cardiomyocytes. (A) Representative images of a heart section from an EGFP-bone marrow-chimeric CreLacZ mouse at 1 month after myocardial infarction (MI). An arrowhead indicates an EGFP and SA-actinin double-positive cardiomyocyte. Serial sections stained for X-gal indicated that the EGFP-positive cardiomyocyte is an X-gal-positive cell. Scale bar, 20 μm. (B) Quantification of the frequency of X-gal and EGFP double-positive bone marrow cell-derived pre-existing cardiomyocytes, X-gal-negative and EGFP-positive bone marrow cell-derived newly formed cardiomyocytes, and X-gal-negative and EGFP-negative newly formed cardiomyocytes derived from endogenous stem cells. One heart section was examined per mouse. The mean from three mice is presented.

It has been reported that BM contains stem cells that transdifferentiate into cardiomyocytes, and the injured myocardium recruits these stem cells [29]. To determine whether X-gal-negative cardiomyocytes were derived from BM stem cells, we created BM-chimeric CreLacZ mice in which 95% of the BM cells had been replaced by cells from transgenic EGFP mice. Chimeric CreLacZ mice were subjected to MI with LIF administration (n = 3). X-gal-positive and EGFP-positive BM cell-derived cardiomyocytes were present at a frequency of 0.18 ± 0.08 cells/mm^2^ (Fig 7A and 7B); however, X-gal-negative and EGFP-positive BM cell-derived newly formed cardiomyocytes were not identified (Fig 7B). X-gal-negative and EGFP-negative (newly generated from resident CPCs or CSCs) cardiomyocytes were present at a frequency of 10.14 ± 3.74 cells/mm^2^. These findings suggest that a fraction of BM-derived cells replenish cardiomyocytes through transdifferentiation or fusion under normal conditions; however, they do not strongly contribute to the generation of newly formed cardiomyocytes after MI.

## Discussion

In this study, we demonstrated that LIF stimulates the de novo generation of cardiomyocytes. Using a genetic fate-mapping system, we identified more newly formed cardiomyocytes in injured myocardium compared with normal myocardium. These new cardiomyocytes originated from LRCs, which shared the stem cell property of slow cycling with CPCs and CSCs in adult mice. Increased blood LIF levels following LIF cDNA transfection induced cell cycle reentry of CSPs, expansion of CSCs, and an increase in the number of new cardiomyocytes concomitant with a decrease in the size of the MI scar.

Several recent reports have suggested that the mammalian heart possesses some capacity for regeneration; thus, there has been substantial interest in elucidating the cell types involved in natural cardiomyocyte renewal and determining whether cardiac injury enhances this regeneration [7,30]. Genetic fate-mapping is advantageous for tracing the fate of cells of a specific lineage. Using MerCreMer mice along with a lineage labeling ZEG strain (MerCreMer-ZEG), Hsieh et al. previously demonstrated that the percentage of labeled pre-existing cardiomyocytes decreased following injury as a result of a dilution effect by newly formed unlabeled cardiomyocytes [31]. However, because only 80% of the cardiomyocytes were initially labeled in the MerCreMer-ZEG system, it remained unclear whether the new cardiomyocytes developed from the non-cardiomyocyte progenitor population or pre-existing, unlabeled cardiomyocytes. In the MerCreMer mice used in this study, more than 99.9% of the working cardiomyocytes were initially labeled following pulsed tamoxifen injection, which enabled us to precisely detect the newly formed working cardiomyocytes derived from unlabeled non-cardiomyocytes.

To examine the stem cell characteristics of the originators of X-gal-negative newly formed cardiomyocytes, we applied the LRC strategy to CreLacZ mice. The presence of LRCs in X-gal-negative cardiomyocytes suggested that some of them originated from slow cycling cells. Furthermore, we demonstrated that CSPs and pGATA4- or Nkx2.5-positive/SA-actinin-negative cells included LRCs in the adult stage. The CSPs belong to the CSC population and have been reported to be quiescent in the normal adult heart [27]. pGATA4-positive or Nkx2.5-positive/SA-actinin-negative cells represent CPCs, which arise from CSCs following asymmetric cell division and maintain a low cell division rate in the normal adult heart [32]. These findings suggest that injury-induced cardiomyocyte regeneration depends, in part, on dormant stem or progenitor cells. Because specific markers that exclusively label CSCs have not yet been identified, we could not directly trace the fate of the CSCs. Uchida et al. recently established transgenic mice in which the doxycycline-dependent expression of Cre recombinase induced permanent activation of reporter genes in cells expressing Sca-1 [33]. The authors demonstrated that a subpopulation of cardiomyocytes was derived from adult Sca-1-positive cells, and both MI and pressure-induced cardiac hypertrophy modestly stimulated the commitment of Sca-1-positive cells to cardiomyocyte differentiation, supporting our findings in terms of endogenous regeneration from non-cardiomyocytes.

Our findings do not exclude the possibility that differentiated cardiomyocytes contribute to renewal. In general, it is accepted that dedifferentiation and the subsequent proliferation of pre-existing cardiomyocytes is the dominant mechanism underlying zebrafish heart regeneration [34,35]. The neonatal mouse heart regenerates myocardium following resection, which is similar to zebrafish [36]. Senyo et al. recently reported that even in the adult mouse heart, pre-existing cardiomyocytes are the dominant source of cardiomyocyte replacement in normal and MI hearts [37]. It is conceivable that the mammalian heart utilizes two distinct cell sources, including stem/progenitor cells and dividing cardiomyocytes. Moreover, BM cells may also potentially target the heart and differentiate into cardiomyocytes [38]. Our results in EGFP-BM-chimeric CreLacZ mice indicated that circulating BM cells did not differentiate for as long as 4 weeks after MI. However, BM-derived EGFP-positive cells were identified as pre-existing cardiomyocytes, which suggests that circulating BM cells may partially contribute to homeostatic cardiomyocyte renewal.

The secretion of factors with paracrine effects by transplanted cells is thought to underlie the beneficial effects of cardiac cell therapy [39]. Using the MerCreMer-ZEG system, Loffredo et al. reported that the injection of BM-derived c-Kit-positive cells into the infarcted heart stimulated cardiomyocyte renewal from endogenous CPCs or unlabeled cardiomyocytes [40]. We demonstrated that MI-stimulated non-cardiomyocyte-derived regeneration is biased to the MI area compared with the MI remote area. In addition, a slight increase in X-gal-negative cardiomyocytes in the MI remote area was identified compared with the sham mice. These findings support the notion that cytokines secreted after inflammation may enhance the regeneration process [41]. Therefore, it is crucial to examine the paracrine factors that stimulate de novo cardiomyocyte regeneration. Our results suggest that the administration of LIF regenerates a substantial number of cardiomyocytes (approximately 1.96 ± 3.33 × 10^4^ cardiomyocytes per heart) and may represent a potent therapeutic strategy for heart failure. The hemodynamic load is increased in the MI area; thus, cardiomyocyte renewal in the MI area has more beneficial effects on the heart. In addition, cardiomyocytes work as paracrine factor-producing cells [42], and regenerated cardiomyocytes are expected to have protective effects on pre-existing cardiomyocytes.

The mechanisms through which LIF affects CSCs and CPCs remain elusive. We demonstrated that LIF promoted injury-induced nucleoside analog (BrdU and EdU) incorporation in X-gal-negative cardiomyocytes, CSCs (CSPs) and CPCs. In agreement with these findings, LIF increased the number of Ki67-positive CSPs after MI and tended to increase the number of Ki67-positive and Nkx2.5-positive CPCs, suggesting that LIF promoted cell proliferation in the early stage of new cardiomyocyte differentiation. These findings are reasonable because cardiomyocyte differentiation without CSC or CPC division causes stem or progenitor cell exhaustion. Several studies have investigated the critical role of JAK-STAT signaling in cardiac differentiation. Foshay et al. reported that the loss of function of JAK2 or STAT3 suppressed cardiac differentiation of mouse ES cells, and the gain of function of JAK2 enhanced cardiac differentiation [43]. Furthermore, Rajasingh et al. reported that LIF and BMP-2 synergistically induced mouse ES cell differentiation into cardiomyocytes via STAT3 signaling [44]. Synder et al. demonstrated that STAT3 binds constitutively to the Nkx-2.5 promoter and inducibly to Tbx5, as well as GATA4 promoters in response to LIF treatment, enhancing the expression of these cardiac transcription factors in P19CL6 embryonic carcinoma (EC) cells [45]. These reports did not specify the stage of LIF action in the cardiomyocyte lineage hierarchy of ES or EC cells; nevertheless, the LIF-JAK-STAT pathway may promote cardiomyogenic differentiation of heart field-specific progenitors that resemble CSCs or CPCs. Our immunocytochemical and flow cytometry analyses demonstrated that LIF phosphorylates STAT3 in CSPs both in vivo and in vitro, which suggests that the JAK-STAT pathway may be involved in both the proliferation and differentiation of CSCs. However, LIF also increased the frequency of p-Akt- and p-ERK-positive CSPs in vivo and in vitro (S5 Fig). Further elucidation of the contribution of each pathway to CSC proliferation and differentiation is necessary.

We demonstrated that CSPs express LIFRs; however, LIF may indirectly affect CSCs or CPCs via other cells in vivo. Shih et al. reported that LIF binding to its receptor on murine stromal cells led to the production of a human hematopoietic stem cell expansion factor [46]. Two other groups reported that LIF enhanced mast cell growth and dorsal root ganglion neuronal survival in fibroblast co-cultures [47,48]. In the heart, both cardiomyocytes and fibroblasts express LIFRs and secrete LIF proteins; thus, LIF acts as a mediator of fibroblast−myocyte crosstalk [49] and may mediate communication between CSCs/CPCs and various cells in the myocardium. Furthermore, it has been reported that LIF is chemotactic for inflammatory cells and stimulates pro-inflammatory cytokines in various inflammatory disorders such as rheumatoid arthritis, cutaneous inflammation, and nervous system injury [50]. Although LIF is often upregulated under inflammatory conditions, it plays an anti-inflammatory role by reducing the production of pro-inflammatory cytokines and increasing the production of anti-inflammatory cytokines in endotoxic shock [51]. Moreover, LIF induces the production of regulatory T cells and inhibits T helper 17 cell differentiation [52]. Recently, regulatory T cells have been demonstrated to improve wound healing after MI, and IL-17, a product of T helper 17 cells, causes inflammation-mediated advanced cardiac remodeling [53,54]. Therefore, LIF may protect CSCs and CPCs against the deleterious effects of inflammation.

A secreted factor that may be systemically administered to bolster a cardiac regenerative response is an attractive idea. For example, neuregulin-1, a cardiac growth factor indispensable for cardiac structural maintenance and functional integrity, promotes cardiomyocyte proliferation in the injured heart, and recent clinical trials have demonstrated that neuregulin-1 treatment both improved cardiac function and reversed remodeling [55,56]. A pharmaceutical form of recombinant human LIF has been used in human clinical trials for recurrent implantation failure and chemotherapy-induced peripheral neuropathy [57,58]. LIF treatment was not efficacious in these trials; however, the treatment was well tolerated, and serious adverse events did not increase.

In conclusion, our findings suggest that LIF plays a role in cardiomyocyte regeneration through CSC and CPC proliferation and that it could be used in clinical applications.

## Supporting Information

S1 Fig Cardiomyocyte-specific X-gal Staining and Immunohistochemical Localization of X-gal-negative and Connexin 40-positive Cardiomyocytes in CreLacZ Mice. (A) X-gal staining and hematoxylin−eosin (HE) staining images of a CreLacZ mouse. Following tamoxifen administration, the CreLacZ mouse hearts turned blue after X-gal staining (upper left panel). Scale bar, 1 mm. An HE-stained whole-heart image of a tamoxifen-treated CreLacZ mouse (upper right panel). Scale bar, 1 mm. A magnified image of the stained sample is shown in the lower panel. Scale bar, 20 μm. (B) β-galactosidase expression in cardiomyocytes in a tamoxifen-treated mouse (left) and a non-treated mouse (right) detected with a β-galactosidase antibody (red). The bottom panels show the immunofluorescence images of the same samples co-stained with anti-SA-actinin (green) and anti-laminin (blue) antibodies. Scale bar, 20 μm. (C) Immunofluorescence images (connexin 40, red; anti-SA actinin, green; DAPI, blue) co-stained with X-gal were obtained from four areas (a−d) indicated in the upper panel. Nearly all X-gal-negative cardiomyocytes were connexin 40-positive and were present in the crest of the muscular ventricular septum and endomyocardium, which suggests that these cells belonged to the cardiac conduction system. Scale bars: 1 mm for a whole heart image; 20 μm for the immunofluorescence images.
(TIF)

S2 Fig X-gal Staining of Non-cardiomyocytes in CreLacZ Mice and Comparative Analysis of the Detection of β-galactosidase Activity. (A) Immunofluorescence images of a small artery co-stained with X-gal (smooth muscle actin, green; laminin, red; DAPI, blue), fibroblasts co-stained with X-gal (SA-actinin, green; vimentin, red; DAPI, blue), and capillaries co-stained with X-gal (SA-actinin, green; CD31, red; DAPI, blue). n = 413 smooth muscle cells pooled from three sections from three mice, n = 690 fibroblasts pooled from three sections from three mice, and n = 333 endothelial cells pooled from three sections from three mice. Scale bar, 20 μm. (B) Flow cytometry histograms showing FDG expression in control mice (left) and CreLacZ mice with tamoxifen (right). (C) Representative X-gal and α-MHC double-staining images of FDG-negative cells isolated from the gated area. An arrow indicates an α-MHC-positive and X-gal-positive cardiomyocyte and an arrowhead indicates an α-MHC-negative and X-gal-negative non-cardiomyocyte. Scale bar, 20 μm. (D) Frequency tables of two variables, including positivity or negativity of X-gal or α-MHC staining, in which each cell represents the cell number.
(TIF)

S3 Fig CSP Cells were Isolated from Cardiac Cells using Flow Cytometric Sorting. CSP cells were characterized by the ability to efflux Hoechst dye, and treatment with verapamil inhibited the efflux.
(TIF)

S4 Fig LIF Reduces the Scar Area and Improves Cardiac Function After MI. (A) LIF treatment attenuated the area of fibrosis. Representative image of Masson’s trichrome staining of the hearts of phosphate-buffered saline (PBS)-treated and LIF-treated mice are shown in the upper panels. For the calculation, one longitudinal section with the maximum inner radius, which typically reflects the maximum MI area, was analyzed per mouse. An average of values obtained from six PBS- and eight LIF-treated mice is shown in the lower panel. *p < 0.05. (B) Fractional shortening of PBS- (n = 8) and LIF-treated (n = 14) mice. *p < 0.05.
(TIF)

S5 Fig LIF Promotes Nuclear p-ERK and p-Akt Accumulation in Isolated CSP Cells. (A) LIF promotes nuclear p-Akt accumulation in isolated CSPs. Representative images of p-Akt-positive CSPs stained with antibodies to MDR1 (green), p-Akt (red), and DAPI (blue). Scale bar, 10 μm. The bar graph indicates the percentage of p-Akt-positive CSPs among all CSPs (n = 2 per group). (B) LIF promotes nuclear p-ERK accumulation in isolated CSPs. Representative images of p-ERK-positive CSPs stained with antibodies to MDR1 (green), p-ERK (red), and DAPI (blue). Scale bar, 10 μm. The bar graph indicates the percentage of p-ERK-positive CSPs among all CSPs (n = 2 each). (C) Representative images of CSPs stained with antibodies to MDR1 (green), p-Akt (red), and DAPI (blue) isolated from the LIF- and PBS-treated mice at 1 week after MI. p-Akt-positive (upper panel) and -negative (lower panel) CSPs are shown. Scale bar, 10 μm. The bar graph shows the percentage of p-Akt-positive CSPs isolated from LIF- and PBS-treated mice at 1 week after MI (*p < 0.05; n = 3 each). (D) Representative images of CSPs stained with antibodies to MDR1 (green), p-ERK (red), and DAPI (blue). p-ERK-positive (upper panel) and -negative (lower panel) CSPs are shown. Scale bar, 10 μm. The bar graph indicates the percentage of p-ERK-positive CSPs isolated from LIF- and PBS-treated mice at 1 week after MI (*p < 0.05; n = 3 each).
(TIF)

S1 Table M-mode Echocardiographic Analysis before and after Tamoxifen Administration. (DOC)

S2 Table M-mode Echocardiographic Analysis 1 Month after Myocardial Infarction. (DOC)
