# Supplementary figures and images for: Leukemia Inhibitory Factor Enhances Endogenous Cardiomyocyte Regeneration after Myocardial Infarction
Source: PLoS One. 2016 May 26;11(5):e0156562. doi: 10.1371/journal.pone.0156562 (PMC4881916; doi:10.1371/journal.pone.0156562)

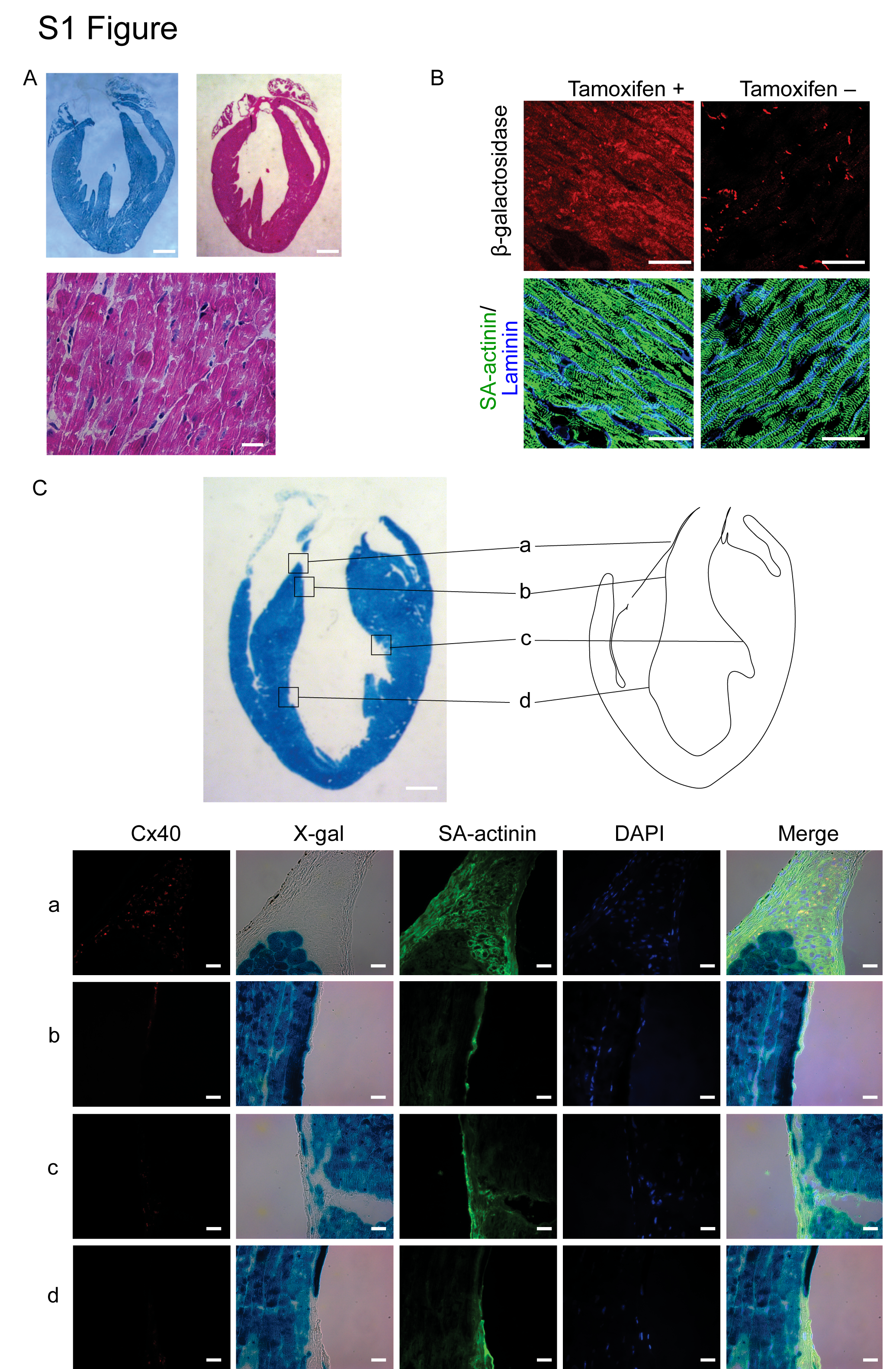

Supplement: S1 Fig — (A) X-gal staining and hematoxylin−eosin (HE) staining images of a CreLacZ mouse. Following tamoxifen administration, the CreLacZ mouse hearts turned blue after X-gal staining (upper left panel). Scale bar, 1 mm. An HE-stained whole-heart image of a tamoxifen-treated CreLacZ mouse (upper right panel). Scale bar, 1 mm. A magnified image of the stained sample is shown in the lower panel. Scale bar, 20 μm. (B) β-galactosidase expression in cardiomyocytes in a tamoxifen-treated mouse (left) and a non-treated mouse (right) detected with a β-galactosidase antibody (red). The bottom panels show the immunofluorescence images of the same samples co-stained with anti-SA-actinin (green) and anti-laminin (blue) antibodies. Scale bar, 20 μm. (C) Immunofluorescence images (connexin 40, red; anti-SA actinin, green; DAPI, blue) co-stained with X-gal were obtained from four areas (a−d) indicated in the upper panel. Nearly all X-gal-negative cardiomyocytes were connexin 40-positive and were present in the crest of the muscular ventricular septum and endomyocardium, which suggests that these cells belonged to the cardiac conduction system. Scale bars: 1 mm for a whole heart image; 20 μm for the immunofluorescence images. (TIF) [file pone.0156562.s001.tif]

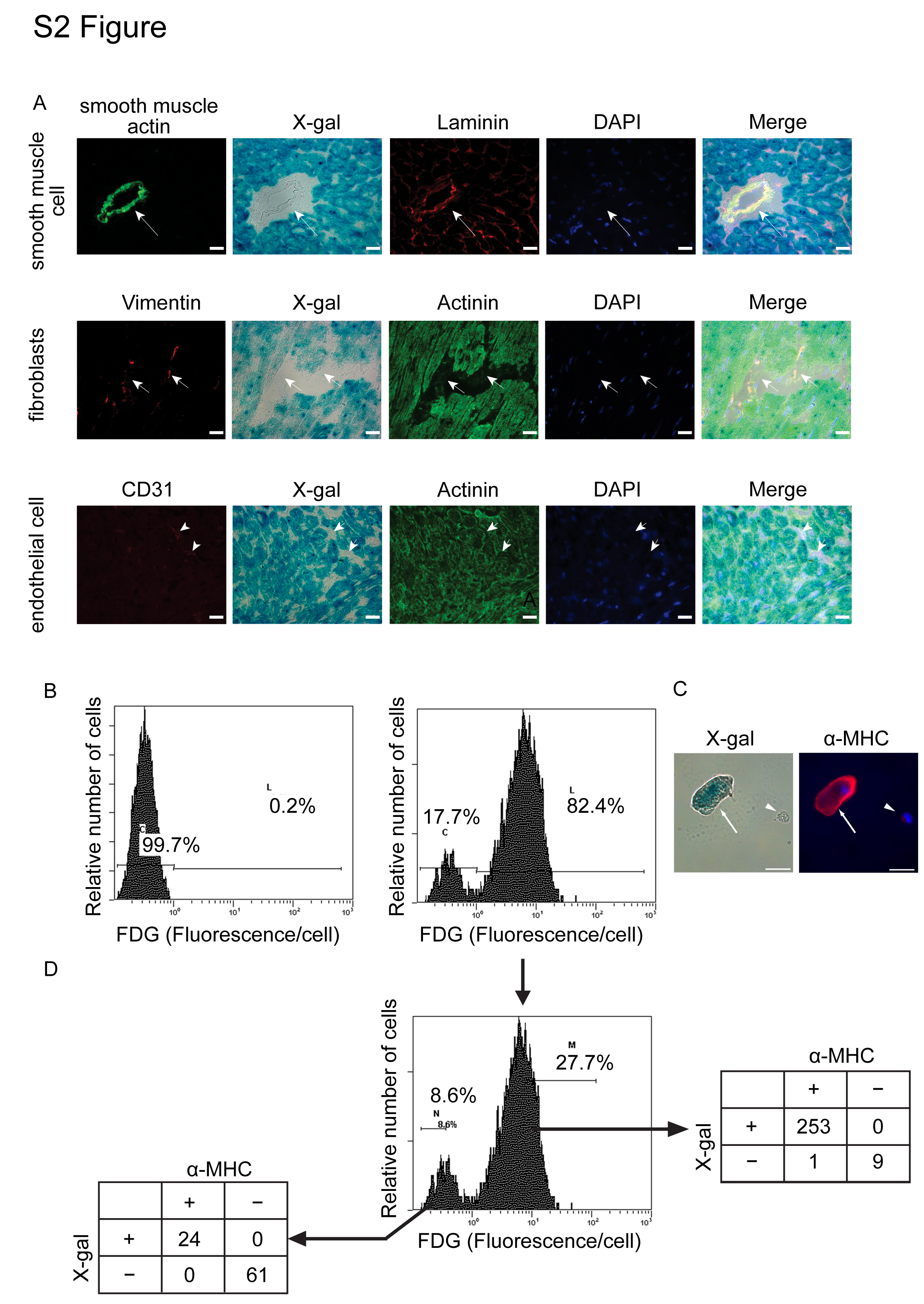

Supplement: S2 Fig — (A) Immunofluorescence images of a small artery co-stained with X-gal (smooth muscle actin, green; laminin, red; DAPI, blue), fibroblasts co-stained with X-gal (SA-actinin, green; vimentin, red; DAPI, blue), and capillaries co-stained with X-gal (SA-actinin, green; CD31, red; DAPI, blue). n = 413 smooth muscle cells pooled from three sections from three mice, n = 690 fibroblasts pooled from three sections from three mice, and n = 333 endothelial cells pooled from three sections from three mice. Scale bar, 20 μm. (B) Flow cytometry histograms showing FDG expression in control mice (left) and CreLacZ mice with tamoxifen (right). (C) Representative X-gal and α-MHC double-staining images of FDG-negative cells isolated from the gated area. An arrow indicates an α-MHC-positive and X-gal-positive cardiomyocyte and an arrowhead indicates an α-MHC-negative and X-gal-negative non-cardiomyocyte. Scale bar, 20 μm. (D) Frequency tables of two variables, including positivity or negativity of X-gal or α-MHC staining, in which each cell represents the cell number. (TIF) [file pone.0156562.s002.tif]

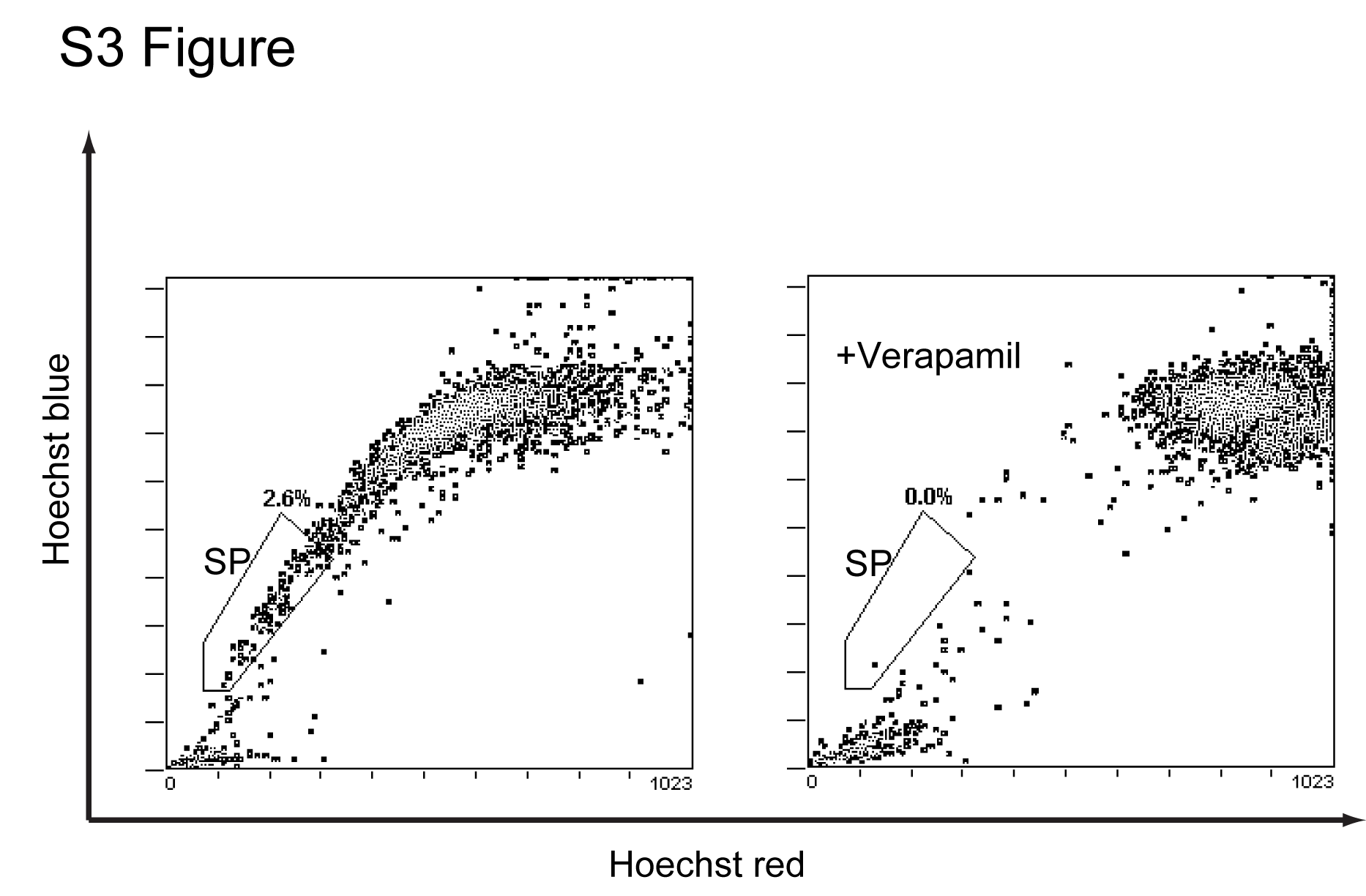

Supplement: S3 Fig — CSP cells were characterized by the ability to efflux Hoechst dye, and treatment with verapamil inhibited the efflux. (TIF) [file pone.0156562.s003.tif]

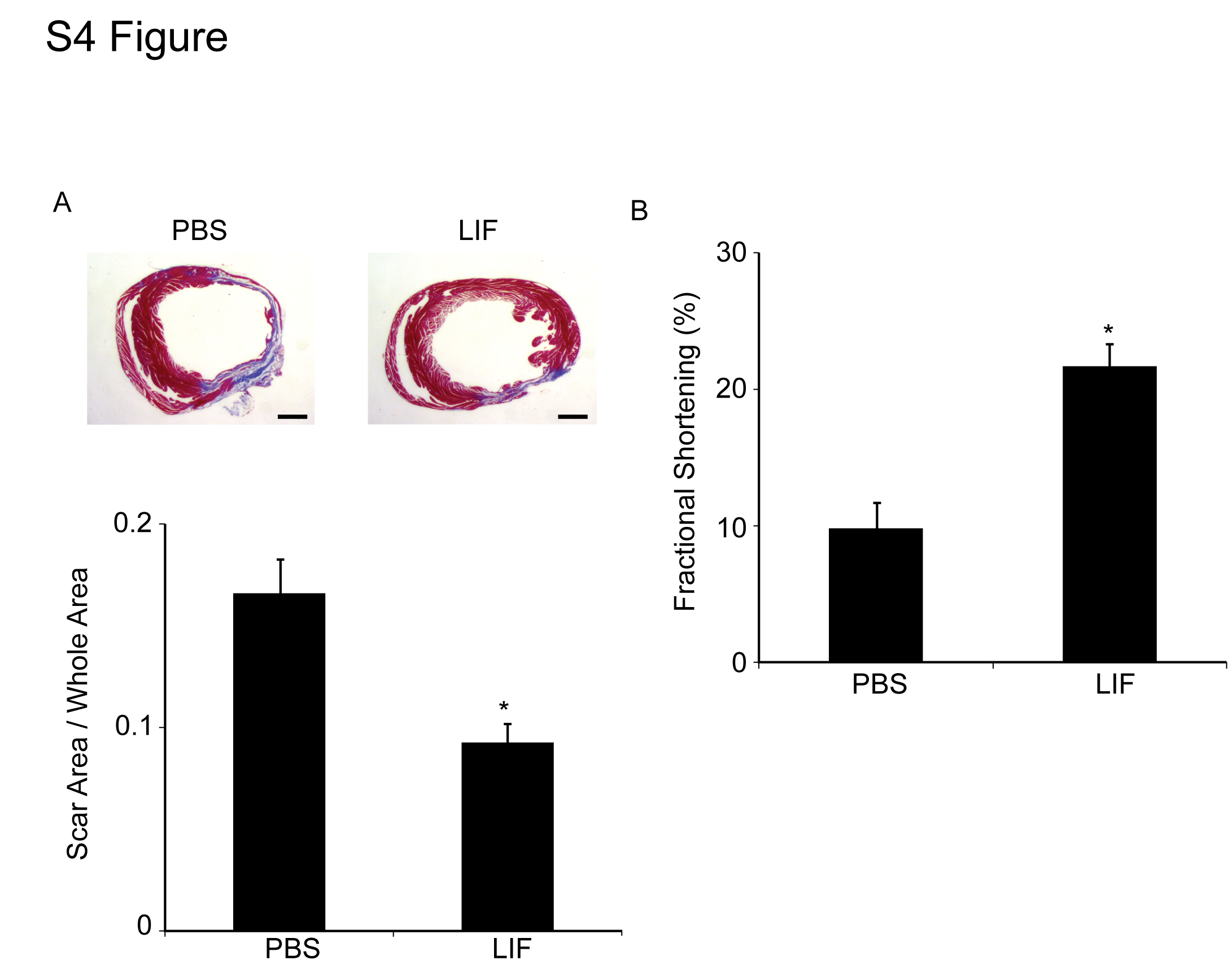

Supplement: S4 Fig — (A) LIF treatment attenuated the area of fibrosis. Representative image of Masson’s trichrome staining of the hearts of phosphate-buffered saline (PBS)-treated and LIF-treated mice are shown in the upper panels. For the calculation, one longitudinal section with the maximum inner radius, which typically reflects the maximum MI area, was analyzed per mouse. An average of values obtained from six PBS- and eight LIF-treated mice is shown in the lower panel. *p < 0.05. (B) Fractional shortening of PBS- (n = 8) and LIF-treated (n = 14) mice. *p < 0.05. (TIF) [file pone.0156562.s004.tif]

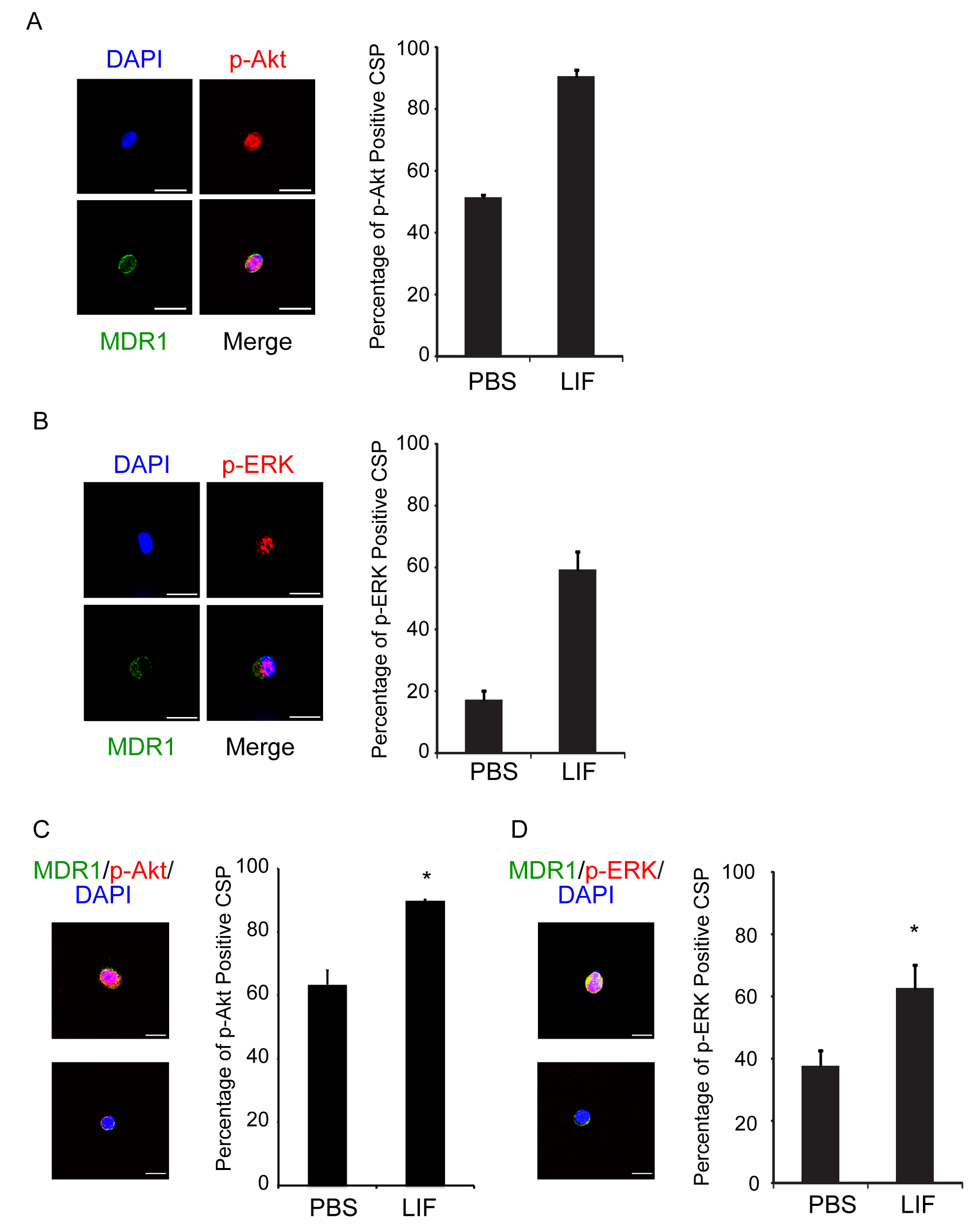

Supplement: S5 Fig — (A) LIF promotes nuclear p-Akt accumulation in isolated CSPs. Representative images of p-Akt-positive CSPs stained with antibodies to MDR1 (green), p-Akt (red), and DAPI (blue). Scale bar, 10 μm. The bar graph indicates the percentage of p-Akt-positive CSPs among all CSPs (n = 2 per group). (B) LIF promotes nuclear p-ERK accumulation in isolated CSPs. Representative images of p-ERK-positive CSPs stained with antibodies to MDR1 (green), p-ERK (red), and DAPI (blue). Scale bar, 10 μm. The bar graph indicates the percentage of p-ERK-positive CSPs among all CSPs (n = 2 each). (C) Representative images of CSPs stained with antibodies to MDR1 (green), p-Akt (red), and DAPI (blue) isolated from the LIF- and PBS-treated mice at 1 week after MI. p-Akt-positive (upper panel) and -negative (lower panel) CSPs are shown. Scale bar, 10 μm. The bar graph shows the percentage of p-Akt-positive CSPs isolated from LIF- and PBS-treated mice at 1 week after MI (*p < 0.05; n = 3 each). (D) Representative images of CSPs stained with antibodies to MDR1 (green), p-ERK (red), and DAPI (blue). p-ERK-positive (upper panel) and -negative (lower panel) CSPs are shown. Scale bar, 10 μm. The bar graph indicates the percentage of p-ERK-positive CSPs isolated from LIF- and PBS-treated mice at 1 week after MI (*p < 0.05; n = 3 each). (TIF) [file pone.0156562.s005.tif]
