# Supplementary material for: Leukemia Inhibitory Factor Enhances Endogenous Cardiomyocyte Regeneration after Myocardial Infarction
Source: PLoS One. 2016 May 26;11(5):e0156562. doi: 10.1371/journal.pone.0156562 (PMC4881916; doi:10.1371/journal.pone.0156562)
Supplement: S1 Table — (DOC) [file pone.0156562.s006.doc]

**S1 Table.** M-mode Echocardiographic Analysis before and after Tamoxifen Administration

|  | Nontransgenic | Pre-tamoxifen | Post-tamoxifen |
| --- | --- | --- | --- |
| Heart Rate, bpm | 649.8 ± 10.1 | 613.6 ± 13.0 | 619.3 ± 9.4 |
| IVSTd, mm | 0.91 ± 0.03 | 0.85 ± 0.02 | 0.92 ± 0.02 |
| LVPWTd,mm | 0.84 ± 0.04 | 0.82 ± 0.07 | 0.83 ± 0.05 |
| LVIDd, mm | 3.18 ± 0.14 | 3.31 ± 0.14 | 3.31 ± 0.13 |
| LVIDs, mm | 1.62 ± 0.12 | 1.62 ± 0.12 | 1.66 ± 0.10 |
| FS, % | 49.3 ± 2.5 | 51.3 ± 1.8 | 50.1 ± 1.4 |

Data are expressed as means ± s.e.m (n = 5 each). IVSTd, interventricular septum thickness; LVPWTd, LV posterior wall thickness; LVIDd and LVIDs, LV internal dimensions at end diastole and end systole, respectively; and FS, fractional shortening. No significant difference in same parameters between nontransgenic and CreLacz mice was observed by the two-tailed Student’s *t*-test.
