# Supplementary material for: Leukemia Inhibitory Factor Enhances Endogenous Cardiomyocyte Regeneration after Myocardial Infarction
Source: PLoS One. 2016 May 26;11(5):e0156562. doi: 10.1371/journal.pone.0156562 (PMC4881916; doi:10.1371/journal.pone.0156562)
Supplement: S2 Table — (DOC) [file pone.0156562.s007.doc]

**S2 Table. M-mode Echocardiographic Analysis after 1 Month of Mycardial Infarction**

|  | PBS | LIF |
| --- | --- | --- |
| N | 8 | 14 |
| IVSTd, mm | 0.50 ± 0.04 | 0.75 ± 0.14* |
| LVPWTd,mm | 0.65 ± 0.06 | 0.82 ± 0.15* |
| LVIDd, mm | 5.34 ± 0.20 | 4.79 ± 0.62 |
| LVIDs, mm | 4.81 ± 0.26 | 3.77 ± 0.75* |
| FS, % | 10.4 ± 1.8 | 21.9 ± 1.7* |

Data are expressed as means ± s.e.m. Tx, tamoxifen; IVSTd, interventricular septum thickness; LVPWTd, LV posterior wall thickness; LVIDd and LVIDs, LV internal dimensions at end diastole and end systole, respectively; and FS, fractional shortening.

*p < 0.05 vs MI with PBS injection.
